# The Impact Mechanism of Inter‐Basin Water Transfer on Fish Assemblages Was Revealed Using a Model of Terminal Reservoirs

**DOI:** 10.1002/ece3.72459

**Published:** 2025-11-02

**Authors:** Zhenhao Cheng, Anxiang Wang, Yujing Cui, Lei Gao, Fei Cheng, Fengyue Shu, Songguang Xie

**Affiliations:** ^1^ School of Life Sciences Qufu Normal University Qufu China; ^2^ Key Laboratory of Freshwater Fish Reproduction and Development (Ministry of Education), College of Fisheries Southwest University Chongqing China; ^3^ Huaihe River and Xiaoqinghe River Basin Water Conservancy Management and Service Center of Shandong Province Water Resources Department of Shandong Province Jinan China; ^4^ Yangtze River Fisheries Research Institute Chinese Academy of Fishery Sciences Wuhan China; ^5^ School of Marine Biology and Fisheries Hainan University Haikou China

**Keywords:** assembly process, diversity index, fish assemblage, newly water body, water transfer

## Abstract

Understanding the impact mechanism of inter‐basin water transfer projects (IBWTs) on fish assemblages is critical for conserving aquatic ecosystems and managing water transfer. However, direct evidence of the impact mechanism of IBWTs on fish assemblages is absent. Fish assemblages in newly built terminal reservoirs originate and assemble with diverted individuals through water transfer in IBWTs, providing a valuable model for mechanistic research. This study sampled fish quarterly using a combination net in three terminal reservoirs along the Eastern Route of the South‐to‐North Water Transfer Project (ER‐SNWTP) from July 2021 to May 2022. We investigated assemblage composition and structure, calculated alpha and beta diversity indices, and analyzed the effects of environmental factors on fish assemblages. A total of 35 species were collected and indicated which species could be diverted accompanying water transfers along the ER‐SNWTP. Fish assemblages were dominated by eurytopic species, but showed clear seasonal differences. The distance from Dongping Lake, operation time of the reservoirs, and water quality affected diversity indices, composition and structure of the fish assemblages in the reservoirs. Fish assemblages in the terminal reservoirs were unstable and periodically disturbed by the water transfers of the ER‐SNWTP. Our results indicate that fish diverted by water transfer are likely to be the main cause of the impact of IBWTs on fish assemblages, and the distance and number of water transfers, and nutrient loading of diverted water are crucial factors affecting the impact of IBWTs. Our study provides a theoretical understanding of the ecological impacts of IBWTs by introducing fish and periodically disturbing fish assemblage through water transfer and highlights the necessity of scheming transfer programs to avoid overlapping with the spawning peaks of some fish with special guilds, such as small‐sized fish. In terminal reservoirs, the fish assemblages are not and may have no possibility to reach a stability like a natural one, and then their management should be oriented toward water quality regulation, such as rational harvesting and replenishment of biomanipulation fish to enhance their capacity for water quality regulation.

## Graphical abstract

Using three terminal reservoirs of an inter‐basin water transfer project (IBWT) as a model system, our study revealed an impact mechanism of IBWTs on fish assemblages. Our study found an underestimated effect of IBWTs on fish assemblages through diverted fish accompanying periodic water transfer, and the distance of water transfer, reservoir operation time, and water quality are crucial factors affecting the impact of IBW Ts. In terminal reservoirs, the fish assemblages are unlikely to reach a natural stability, and then their management should be oriented toward water quality regulation.

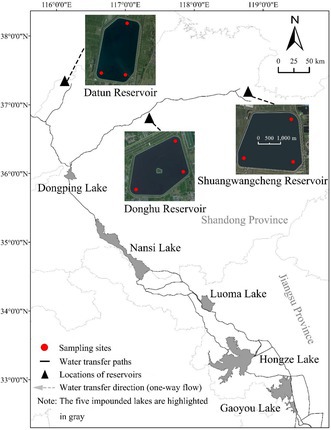

## 1 Introduction

Inter‐basin water transfer projects (IBWTs) have been used globally to regulate and alleviate the regional scarcity of water resources (Ghassemi and White 2007; Vitule et al. 2015; Daga et al. 2020). Worldwide, over 170 IBWTs are operated, with approximately 25% of global freshwater resources being diverted (Schmidt et al. 2020). In China, more than 50 billion m^3^ of water from the Yangtze River had been diverted through the South‐to‐North Water Transfer Project (SNWTP) from 2014 to 2021, which was beneficial to 150 million people in water‐scarce regions (The State Council of the People's Republic of China 2022). IBWTs have made substantial achievements in improving civilian living quality and optimizing economic development patterns and more projects are underway to achieve sustainable development in water‐scarce regions with rising populations (Vitule et al. 2015; Purvis and Dinar 2020; Sun et al. 2021). However, IBWTs may trigger some ecological risks in water bodies along diversion paths and recipient regions (Feng et al. 2018; Daga et al. 2020; Schmidt et al. 2020).

The negative ecological impacts associated with IBWTs have been extensively recorded, especially in fish (Swift et al. 2014; Silveira et al. 2017; Ramos et al. 2021). Numerous fish invasions accompanied by water diversions have been reported globally over the past 20 years (Grant et al. 2012; Gallardo and Aldridge 2018; Qin et al. 2023). IBWTs facilitate the dispersal of fish with specific traits, and thus alter the composition of local fish assemblages (Grant et al. 2012; Yan et al. 2025). After fish have entered the water bodies of IBWTs, some studies have concluded that biotic homogenization of fish assemblages has increased among waters in an IBWT (Guo et al. 2020; Liu et al. 2022). Biotic homogenization is a process of changes in the species composition, characterized by increasing similarity and simplified structure of fish assemblages across different water bodies (McKinney and Lockwood 1999). However, information on the homogenization process, such as characteristics and impact factors, is scarce for fish assemblages in the water bodies of IBWTs.

Only fish with specific traits are likely to be diverted easily and to be assembled into the local assemblage, which can result in the fish homogenization of the water bodies of IBWTs (Schmidt et al. 2020). First, these species should have a higher chance of dispersal through water transfer. Schmidt et al. (2020) reviewed the potential factors promoting fish movement in IBWTs and scored a higher likelihood of dispersal to groups/species with specific traits such as small‐sized body, migratory, generalist, and pelagic habitat preferences. Second, these fishes should have higher colonization potential to adapt to abiotic conditions and achieve interspecific resource differentiation (Qin et al. 2020). Because these diverted species will be filtered by abiotic conditions and interact with biotic factors in a new habitat after their introduction (Burgad et al. 2023; Qin et al. 2023). Eurytopic and omnivorous fish have wider niche breadth and may be more successful in the dispersal and establishment of populations in water bodies of IBWTs (Gallardo and Aldridge 2018; van Treeck et al. 2020). Distinct changes in local fish assemblages were observed among water bodies of IBWTs (Daga et al. 2020; Schmidt et al. 2020; Liu, Brosse, et al. 2023). These differences in the homogenizing process may be influenced by the fauna and trait composition of local fish assemblages, the modes and locations of IBWTs, and the local water environment, suggesting that further mechanistic research is needed (Cheimonopoulou et al. 2011; Guo et al. 2020).

Furthermore, research on the impact mechanism of IBWTs needs to highlight periodic characteristics of water transfers. The water transfer is usually periodic and implemented annually in IBWTs, such as the Eastern Route of SNWTP, China (ER‐SNWTP), Potter Valley Project, America (Ma et al. 2020; Yan et al. 2025). Fish may be periodically diverted by water transfers, which can disturb local fish assemblages along IBWTs and substantially hinder the stability of the established assemblages (Yang et al. 2008; Yan et al. 2025). Unpredictable and highly influential events may lead to stochastic assembly processes in fish assemblages, which indicate unstable states and randomness of fish assemblage development (Bernardo et al. 2003). For instance, research in Brazilian reservoirs confirmed that persistent disturbances caused by dam operation patterns and water level fluctuations substantially inhibit the development of fish assemblages toward stable states (Agostinho et al. 2016). Subject to periodical disturbance of water transfer, fish species with wider niche breadth, such as eurytopic and omnivorous species, may have better competitive advantages and thus become dominant in assemblages. Thereby, fish periodically diverted by water transfer may be a key factor shaping fish assemblages in water bodies of IBWTs. For the conservation and utilization of fish in the waters of IBWTs, their management should also consider the instability of fish assemblages caused by the periodic effects of water transfer.

Overall, existing studies have recorded impacts of IBWTs on fish. However, the impact mechanisms need to be disentangled for designing mitigation strategies for IBWTs. Knowledge of the impacts of IBWTs on fish and their mechanisms is vital for formulating ecological transfer schemes of IBWTs, which are urgently needed. An optimization of the transfer scheme has been proposed to decrease impacts and increase suitable spawning and nursery habitats for Atlantic salmon ( *Salmo salar* L.), based on research in a terminal reservoir of the Kielder water transfer system, England (Gibbins et al. 2002).

However, the impacts of IBWTs are difficult to determine through spatial comparison of alterations in fish assemblages, because the assemblage compositions and structures are set through direct or indirect natural conditions (biotic and abiotic factors), multilayered ecological processes, and anthropogenic disturbances from multiple sources (e.g., water pollution, fishing, and land use) (Brucet et al. 2013; Guo et al. 2019). Moreover, due to the lack of historical data in some water bodies along IBWTs, the impacts of IBWTs are difficult to determine through the temporal comparisons of fish assemblage variations (Liu, Brosse, et al. 2023). A suitable research model is urgently needed to reveal the impacts of IBWTs on fish assemblages and their underlying mechanisms.

Terminal reservoirs, particularly some newly built reservoirs, are allocated to recipient regions, providing a valuable model for revealing the impact of IBWTs on fish. These newly built terminal reservoirs usually are constructions with no connectivity with surrounding water bodies (Yan, Chen, et al. 2023). Detailed information on the water transfer is available for these terminal reservoirs (Dević 2015; Dobbs et al. 2023). The fish assemblage in a newly built reservoir of IBWTs is directly related to fish diverted via the water transfer, which can be inferred from the origin and diverted route of water transfer (Guo et al. 2012; Dobbs et al. 2023; Yan, Chen, et al. 2023). Generally, fish can actively and/or passively enter through water transfer and then assemble in a new terminal reservoir, that is reconfigured by fish of the same origin and delivered via annual water transfer (Agostinho et al. 2008; Yan, Chen, et al. 2023). Small‐sized, migratory, generalist, and pelagic fish species are more likely to disperse through water transfers, and these species tend to be pioneers in fish assemblages in the new terminal reservoirs (Schmidt et al. 2020). Additionally, the terminal reservoirs of IBWTs have standardized management and documented all regulatory events, such as fish release for biomanipulation, during their operating processes, which provides an opportunity to comprehensively understand the effects of anthropogenic disturbances on fish assemblages.

In this study, we used three terminal reservoirs of the ER‐SNWTP with gradient controls in distance and periodicity of the water transfers as a test model. We investigated assemblage compositions and structures, calculated alpha and beta diversity indices, and analyzed the effects of multiple environmental factors on fish assemblages. This study aimed to determine the fundamental information on fish assemblages in the three reservoirs and to reveal the impacts of IBWTs on fish assemblages and their crucial factors. We focused on the effects of diverted fish and periodic disturbance by water transfers on fish assemblages, which have been rarely addressed in previous studies. We hypothesized that: (1) fish in the three terminal reservoirs are diverted from Dongping Lake; assemblage compositions and structures may be characterized by fish with high dispersal potential, such as a small‐sized body, and migratory, generalist; and the distance from the lake may have important effects on the fish composition. (2) With the diversion of diverse guilds of fish by periodic water transfers, the composition and structure of fish assemblages may be extremely unstable, and omnivorous and eurytopic fish are likely to dominate the assemblages. Our results provide a theoretical understanding of the ecological impacts of IBWTs, and shed light on the assembly processes of fish assemblages in new water bodies.

## 2 Materials and Methods

### 2.1 Site Selection and Data Collection

The ER‐SNWTP, which includes many newly built terminal reservoirs, is part of the largest IBWT worldwide and has been in operation since 2013 (Xia et al. 2022). Three new terminal reservoirs were constructed as part of the ER‐SNWTP. These reservoirs are not connected to surrounding water bodies and annually impound diverted water, mainly from Dongping Lake. Reservoirs store and supply diverted water directly to meet the domestic, agricultural, and industrial demands of neighboring regions. Therefore, the fish assemblages in these terminal reservoirs are important for regulating the water quality to achieve the goals of water transfer.

**FIGURE 1:**
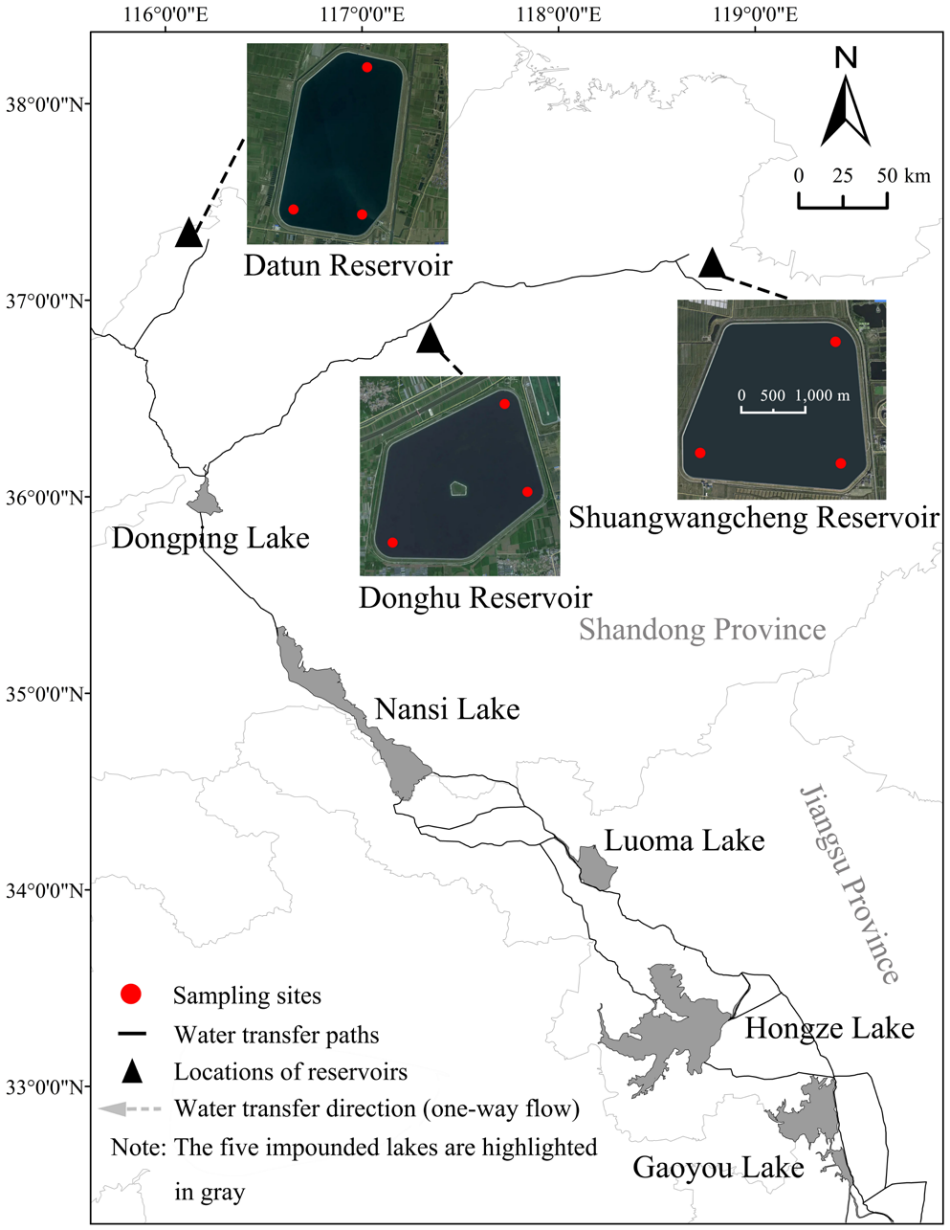
Sketch map of the Eastern Route of the South‐to‐North Water Transfer Project and the distribution of sampling sites in the three terminal reservoirs. Reservoir outline sketches are shown at different scales from those in the remainder of the map.

Three terminal reservoirs of the ER‐SNWTP were selected, Donghu Reservoir (DH, 36°51′54″–36°53′15″ N, 117°18′46″–117°20′28″ E), Datun Reservoir (DT, 37°15′42″–37°17′27″ N, 116°11′13″–116°12′42″ E), and Shuangwangcheng Reservoir (SWC, 37°7′16″–37°8′42″ N, 118°41′41″–118°43′38″ E). These terminal reservoirs were constructed by artificial excavation with geomembranes for horizontal anti‐seepage, reinforced embankments, simple physical structures, and forming basins on the plain. The three terminal reservoirs were similar in terms of regional climate, local environment, and water transfer schemes (Table S1; Figure S1). DH received its first impoundment in December 2020 following its reconstruction, whereas DT and SWC received their first impoundment in November and June 2013, respectively. DH and SWC share a transfer route, whereas DT has its own route. DH and DT are at similar distances from Dongping Lake, whereas the distance of SWC is approximately twice that of the other reservoirs (Table S2; Figure 1). The three reservoirs typically receive water from Dongping Lake from November to May of the following year. Collectively, DH, DT, and SWC formed gradient controls along the ER‐SNWTP based on their distance from Dongping Lake and first impoundment time. Fish assemblages in the reservoirs shared the same origin (Dongping Lake) and mode of introduction. Although there are no specific fish conservation measures at all three reservoirs, unauthorized access and fishing activities have been effectively prevented through perimeter fencing and strict oversight by the management authorities. Therefore, these terminal reservoirs provide an ideal model to establish a direct relationship between variations in fish assemblages and the water transfers of the ER‐SNWTP, which favors revealing the impact mechanism of IBWTs on fish assemblages.

The surveys were conducted in July and October 2021 and January and May 2022 according to the transfer scheme of the ER‐SNWTP from 2021 to 2022. Fish were sampled using a combination net, including multi‐mesh gillnet and trap nets (Guo et al. 2020).

The multi‐mesh gillnet comprised 12 panels with mesh sizes of 5, 6.25, 8, 10, 12.5, 15.5, 19.5, 24, 30, 35, 43, and 55 mm, and then the panels were randomly assembled and maintained in the same order for all multi‐mesh gillnets. Each gillnet was 90 m in length, 2 m in height, and 7.5 m in length for each panel. The trap net with a mesh size of 4 mm, was 18 m in length, 0.45 m in width, and 0.33 m in height, and the mouth openings of the trap were 0.15 m. The trap nets were used without bait.

In each reservoir, we set three sampling sites; each combination net was placed in the littoral zone and exposed for 12 h, from dusk to the following morning (Prchalová et al. 2011; Figure 1). The multi‐mesh gillnet was deployed in a straight line approximately parallel to the shoreline, and the water depth of the sampling sites was approximately 7 m in all reservoirs. Trap nets were dispersed and placed perpendicular to and from the shoreline (Figure 1).

All fish were collected from the combination net, and were identified according to their morphological characteristics, sorted, and counted (Cheng and Zhou 1997; Chen 1998; Ni and Wu 2006). To quantify the variations in fish assemblages among sampling months and reservoirs, the catch number per unit effort (NPUE) and biomass per unit effort (BPUE) were calculated based on each sample using a combination net, following Hinton and Maunder (2004).

Five ecological guilds, including habitat preference, body size, feeding habits, inhabiting water layers, and spawning type, were selected to reflect the different effects of water transfer on fish species with different life history strategies (Feng et al. 2023). Detailed information on the ecological guilds was shown in Table S3, and these guilds were determined for each species based on previous studies (Cheng and Zhou 1997; Chen 1998; Ni and Wu 2006).

The environmental factors included 12 water quality parameters, water transfer distances, and the reservoir operation times (reflecting the periodicity of the water transfer). Water quality parameters were measured before each sampling event (Table S1). The distance of water transfer (DIS), namely, the length of the water diversion canals between the reservoirs and Dongping Lake, was measured using the Bigemap GIS Office (v25.5.0.1) software (Table S2). Because the reservoirs received water diverted from Dongping Lake every year after their first impoundment, the operation times of the reservoirs (ST) represented the water transfer times of each reservoir (Table S2).

### 2.2 Analysis of Assemblage Structure

The dominant species were determined using the relative importance index (IRI) (Pinkas et al. 1971). Non‐metric multidimensional scaling analysis (NMDS) was performed to identify visual differences in the assemblage structure among reservoirs based on Bray–Curtis similarity matrices. Prior to NMDS, raw data were square‐root transformed to reduce the influence of dominant and rare species. The stress coefficients were used to evaluate the reliability of NMDS. The coefficient below 0.1 indicates a good ordination fit with minimal risk of misinterpretation (Clarke 1993). A 3‐dimensional NMDS was finally chosen based on the interpretability of axes, *R* ^2^ of linear fit, stability of solution, and stress value. Permutational multivariate analysis of variance (PERMANOVA) and pairwise tests were conducted to detect differences in the assemblage structure among the reservoirs. NMDS and PERMANOVA were performed using the “vegan” package in R 4.2.2 (Oksanen et al. 2020; R Development Core Team 2020). Similarity percentage analysis (SIMPER) was performed to determine the contribution of each species to the differences in the assemblage structure based on Bray–Curtis dissimilarity indices using Primer 6 (Clarke 1993; Clarke and Gorley 2006).

### 2.3 Analysis of Diversity Indices

A combination of taxonomic, functional, and phylogenetic diversity was used in this study. The alpha taxonomic diversity (TD) of the fish assemblages was assessed using the total number of species (Richness), Shannon–Wiener diversity index (Shannon), Margalef diversity index (Margalef), and Pielou evenness index (Pielou) (Jurasinski et al. 2009). The alpha functional diversity (FD) was quantified using functional richness (FRic), functional evenness (FEve), functional divergence (FDiv), functional dispersion (FDis), and functional redundancy (FRed) indices (Villéger et al. 2008; Ricotta et al. 2016). The functional traits used for FD were selected following Villéger et al. (2017) and are shown in Table S4. They were selected from five categories including food acquisition, mobility, defense against predation, nutrient budget, and reproduction, which are considered to be the main functions that affect ecological roles and services of fish (Liu, Brosse, et al. 2023). Since the true phylogeny comprising all the collected fishes was unavailable, a taxonomic difference index based on classification distance was used as a proxy of the alpha phylogenetic diversity (PD; Liu, Qu, et al. 2023). PD included taxonomic diversity (Δ), taxonomic distinctness (Δ*), average taxonomic distinctness (Δ^+^), and variation in taxonomic distinctness (Ʌ^+^) (Clarke and Warwick 1998, 2001; Schweiger et al. 2008).

The beta diversity of fish assemblages included beta taxonomic (TDβ), beta functional (FDβ), and beta phylogenetic (PDβ) diversity. The overall dissimilarity (sor), turnover component (sim), and nestedness component (sne) for each beta diversity were assessed using the Sorensen dissimilarity matrix (Baselga 2010). Detailed calculations of the beta diversity are available in Liu, Qu, et al. (2023). To calculate the PDβ, a phylogenetic classification tree based on the classification distance was generated as a proxy of the phylogenetic distance, using the phylogeny of Actinopterygian fishes including 31,526 species developed by Rabosky et al. (2018) and Chang et al. (2019).

The “vegan” package and the “FD” package were used to calculate TD and FD, respectively; while the calculation of PD was performed in Primer 6 software (Clarke and Gorley 2006; Laliberté et al. 2015). The “betapart” package was used for TDβ, FDβ, and PDβ analyses (Baselga and Orme 2012). The “fishtree” package was used for the phylogenetic classification tree in PDβ (Chang et al. 2019). All of the aforementioned packages were performed using R version 4.2.2 (R Development Core Team 2020).

### 2.4 Data Analysis

Redundancy analysis (RDA) was conducted to identify the effects of environmental factors on fish assemblages, because the prior result of detrended correspondence analysis showed a first‐axis gradient length of 3.37 (< 4 SD; Legendre and Legendre 2012). Prior to RDA, all environmental factors except pH were log‐transformed to ensure normality and homogeneity. Spearman's correlation coefficients and variance inflation factors were calculated to test for multicollinearity among the environmental factors (Jia et al. 2021). Factors with correlation coefficients > 0.7 (or < −0.7) and variance inflation factors > 10 were eliminated to avoid high collinearity (Figure S2; Jia et al. 2021). Forward selection methods were used to identify key factors in the RDA (*p* < 0.05). The Monte Carlo permutation test was used to test the significance of the environmental factors. The significance level was set at *p* < 0.05.

Analysis of variance (ANOVA) and multiple comparisons were conducted to examine differences in diversity indices among the reservoirs. The Kruskal–Wallis test was used when the normality and homogeneity assumptions of variance were violated. For alpha diversity, multiple linear regressions were performed to analyze the effects of environmental factors, and a forward selection method was used to identify the key explanatory variables in each model. Principal coordinates Analysis (PcoA) was performed on Sorensen dissimilarity matrices derived from overall dissimilarity, turnover, and nestedness components of each beta diversity (TDβ, FDβ, and PDβ) to extract their characteristic vectors as response variables (Liu, Qu, et al. 2023). RDA and variation partitioning analysis were conducted to disentangle drivers of TDβ, FDβ, and PDβ patterns. The predictor variables were first selected from each set of driving factors using the forward selection method in the RDA models. Variation partitioning analysis was performed when two or more complementary sets of predictor variables could be used to explain the variation in the response variables (Peres‐Neto et al. 2006; Blanchet et al. 2008).

All of the aforementioned analyses were carried out using the package “vegan,” “ape,” “corrplot,” and “adespatial” in R 4.2.2 (R Development Core Team 2020).

## 3 Results

### 3.1 Species Composition

A total of 2715 fish were collected from the three reservoirs, which were classified into 35 species, belonging to 4 orders, 8 families, and 30 genera (Table S3). Seasonal variations in species numbers were similar in DH and DT; assemblages of both reservoirs had the lowest species numbers in January, and the highest species numbers occurred in July at DH and in May at DT. Species numbers were lower in SWC with a different pattern of seasonal variation compared with the other two reservoirs.

**FIGURE 2:**
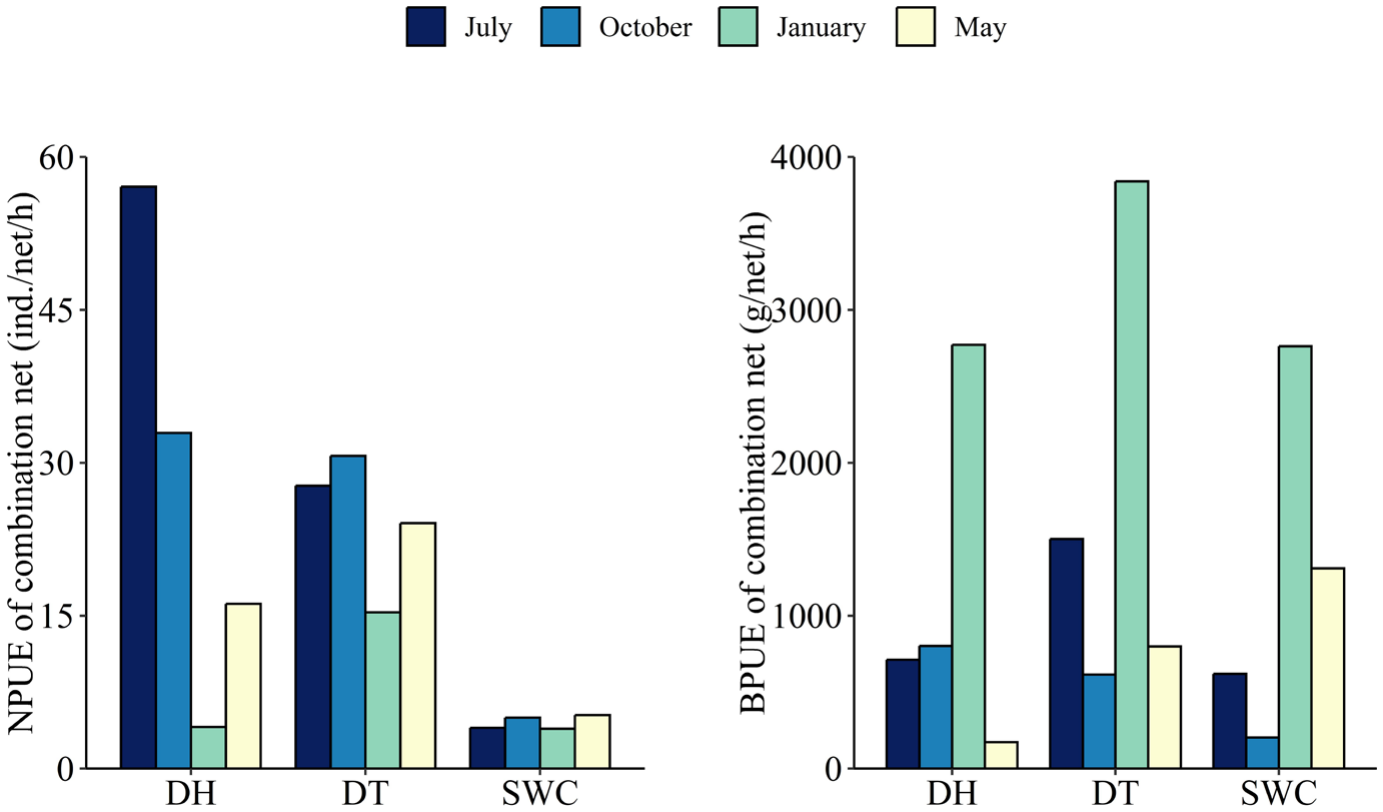
Number per unit effort (NPUE) and biomass per unit effort (BPUE) of fish assemblages in the Donghu (DH), Datun (DT), and Shuangwangcheng (SWC) reservoirs of the Eastern Route of the South‐to‐North Water Transfer Project.

Fish assemblages in the three reservoirs were extremely unstable in terms of abundance and biomass before and after water transfer. Mean NPUE (±SE) was 18.85 ± 4.74 ind./net/h, ranging from 3.92 to 57.08 ind./net/h, and mean BPUE (±SE) was 1342.25 ± 337.39 g/net/h, ranging from 172.96 to 3840.49 g/net/h. NPUE and BPUE varied dramatically between the sampling months in all reservoirs, except for NPUE in SWC (Figure 2). Fewer small‐sized fishes in the assemblages were detected in SWC, which had lower NPUE values and roughly similar BPUE values compared to DH and DT (Figure 2).

**FIGURE 3:**
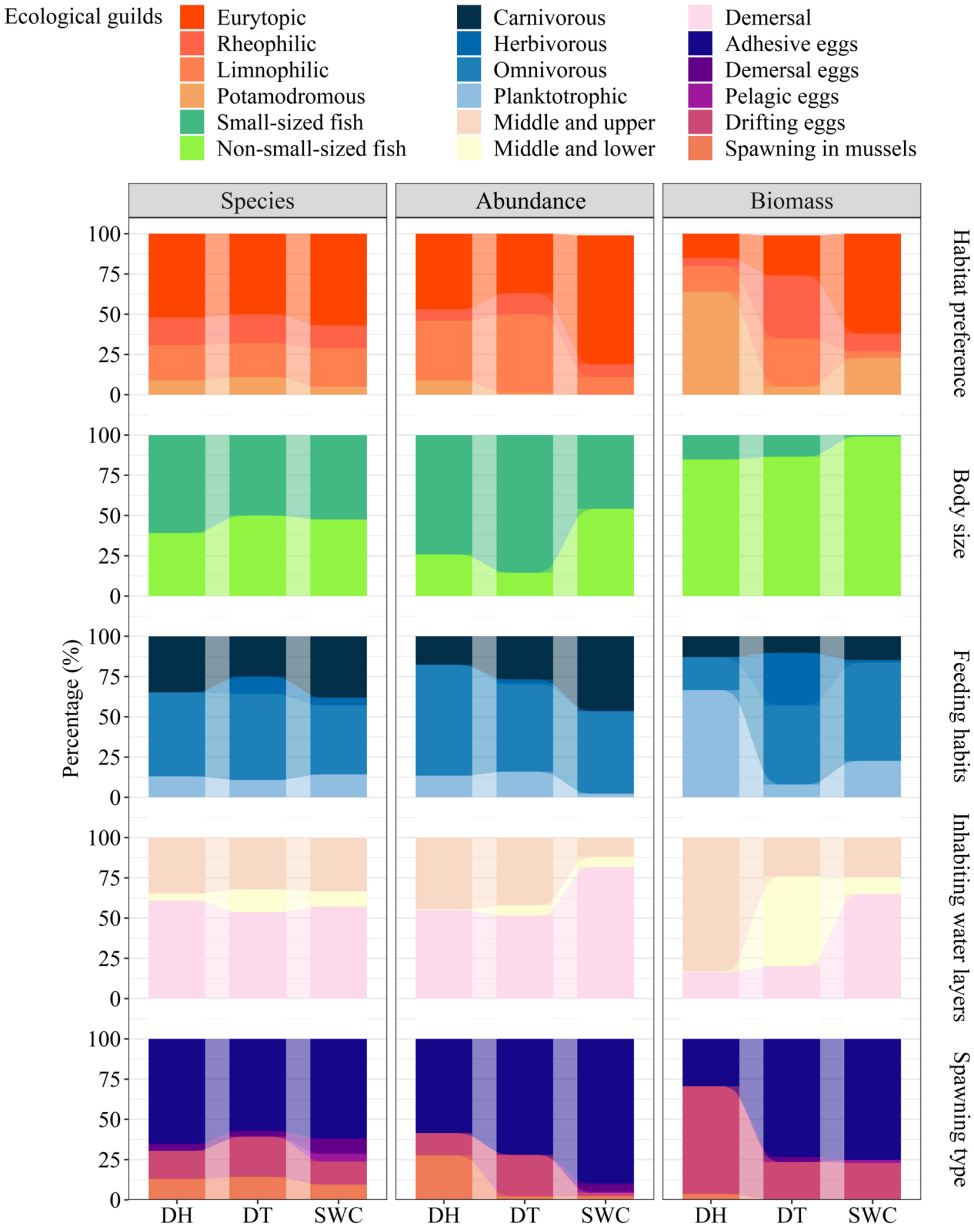
Proportion in ecological guilds of fish assemblages in Donghu (DH), Datun (DT), and Shuangwangcheng (SWC) reservoirs of the Eastern Route of the South‐to‐North Water Transfer Project. Proportions of species number, abundance, and biomass are shown from left to right, and habitat preference, body size, feeding habits, inhabiting water layers, and spawning type are shown from top to bottom.

The dominant guilds of the assemblages in the reservoirs were eurytopic and omnivorous fish, which were evident in species number and abundance; small‐sized fish were comparable in species number, and were dominant in abundance. Both eurytopic and small‐sized fish species accounted for more than 50% of the total species in each reservoir. The proportions of eurytopic fish in terms of species number, abundance, and biomass were highest in SWC, ranging from 57.14% to 80.28%, whereas the proportions of small‐sized fish in terms of abundance were 74.22% and 85.52% in DH and DT, respectively (Figure 3). Among the reservoirs, proportions (±SE) in species number and abundance of omnivorous fish were 49.53% ± 3.36% and 58.07% ± 5.49% (Figure 3).

Except for two species of Gobiidae in DT and SWC, and one species of Bagridae in SWC, all dominant species in the three reservoirs belonged to Cyprinidae (Table S3). The compositions of the dominant species varied greatly among sampling sites and months (Table S3). No dominant species was shared among the three reservoirs. However, shared dominant species were observed between the two reservoirs: *Hemiculter leucisculus* and *Pseudorasbora parva* , two small‐sized fish in DH and DT, *Aristichthys nobilis* in SWC and DH, and *Hemibarbus maculatus* in SWC and DT (Table S3). During all sampling months, only *Carassius auratus* in DT and *H. maculatus* in SWC maintained consistent dominance, whereas no species was consistently dominant in DH (Table S3).

### 3.2 Assemblage Structures

**FIGURE 4:**
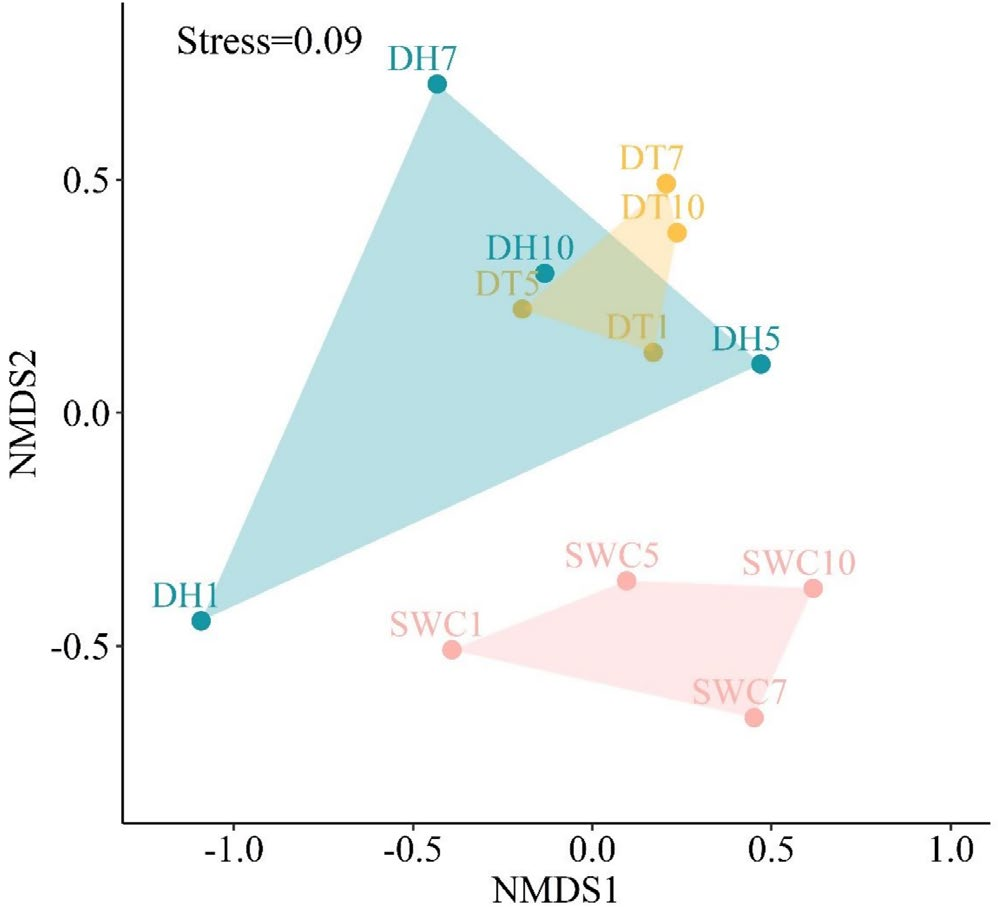
Non‐metric multidimensional scaling analysis (NMDS) on the relative abundance of fish species in Donghu (DH), Datun (DT), and Shuangwangcheng (SWC) reservoirs of the Eastern Route of the South‐to‐North Water Transfer Project. The symbol includes the sampling site and month; for example, DH1 indicates sampling at Donghu Reservoir in January; NMDS was performed with *k* = 3 dimensions (stress = 0.09), the plot shows the projection of the first two axes.

The assemblage structures differed significantly between the three reservoirs (PERMANOVA, *F* = 1.79, *p* < 0.05). Pairwise tests showed that the assemblage structures were significantly different between SWC and DT (*F* = 1.92, *p* < 0.05), roughly different between SWC and DH (*F* = 1.91, *p* = 0.08), and did not differ between DH and DT (*F* = 1.53, *p* > 0.05) (Figure 4, Table S5). Six small‐sized fish were the main contributors to the differences in the assemblage structure between SWC and DT, contributing 50.42% of the dissimilarity (Table S6).

**FIGURE 5:**
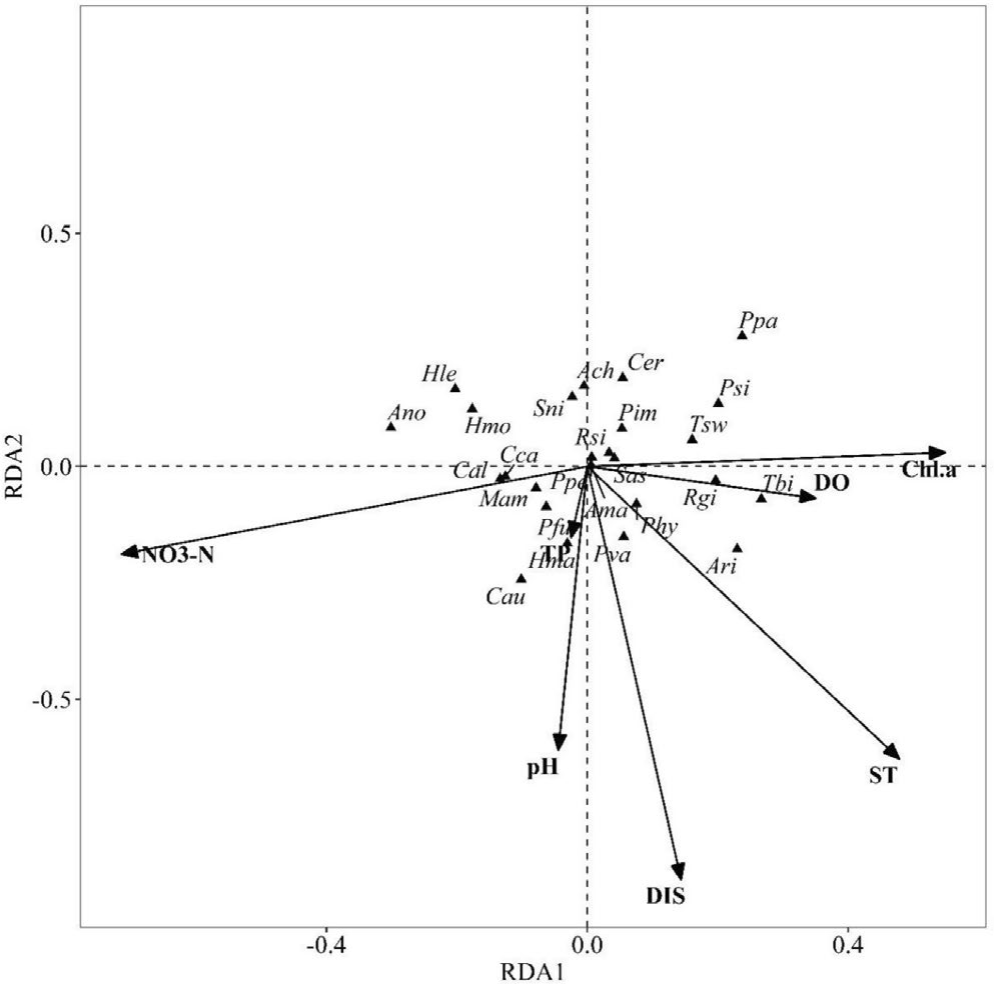
Redundancy analysis (RDA) of species contributing to the dissimilarities of the fish assemblages in the three reservoirs and the environmental factors of the Eastern Route of the South‐to‐North Water Transfer Project.

The first two axes explained 28.41% and 22.32% of the total variation in the RDA model, respectively (adjusted *R*‐squared = 0.15). Results of RDA showed that DIS (*F* = 1.743, *p* < 0.05) and NO_3_‐N (*F* = 1.767, *p* < 0.05) were the key environmental factors affecting the differences in the fish assemblages among the reservoirs. Environmental factors significantly affected the distribution of small‐sized fish, which resulted in most of the differences among the assemblage structures in the three reservoirs. Low abundance of small‐sized fish such as *Sarcocheilichthys nigripinnis* , *Acheilognathus chankaensis* , and *H. leucisculus* was closely linked to long DIS, and high abundance of fish spawning adhesive eggs such as *Culter alburnus* , *Cyprinus carpio* , and *Megalobrama amblycephala* was closely linked to high NO_3_‐N (Figure 5). No significant effects were found between other environmental factors, and differences in fish assemblages among the reservoirs, such as ST (*F* = 1.27, *p* > 0.05), TP (*F* = 1.13, *p* > 0.05), and Chl.a (*F* = 1.34, *p* > 0.05).

See Tables S1 and S3 for codes of environmental factors and fish species.

### 3.3 Diversity Patterns

**FIGURE 6:**
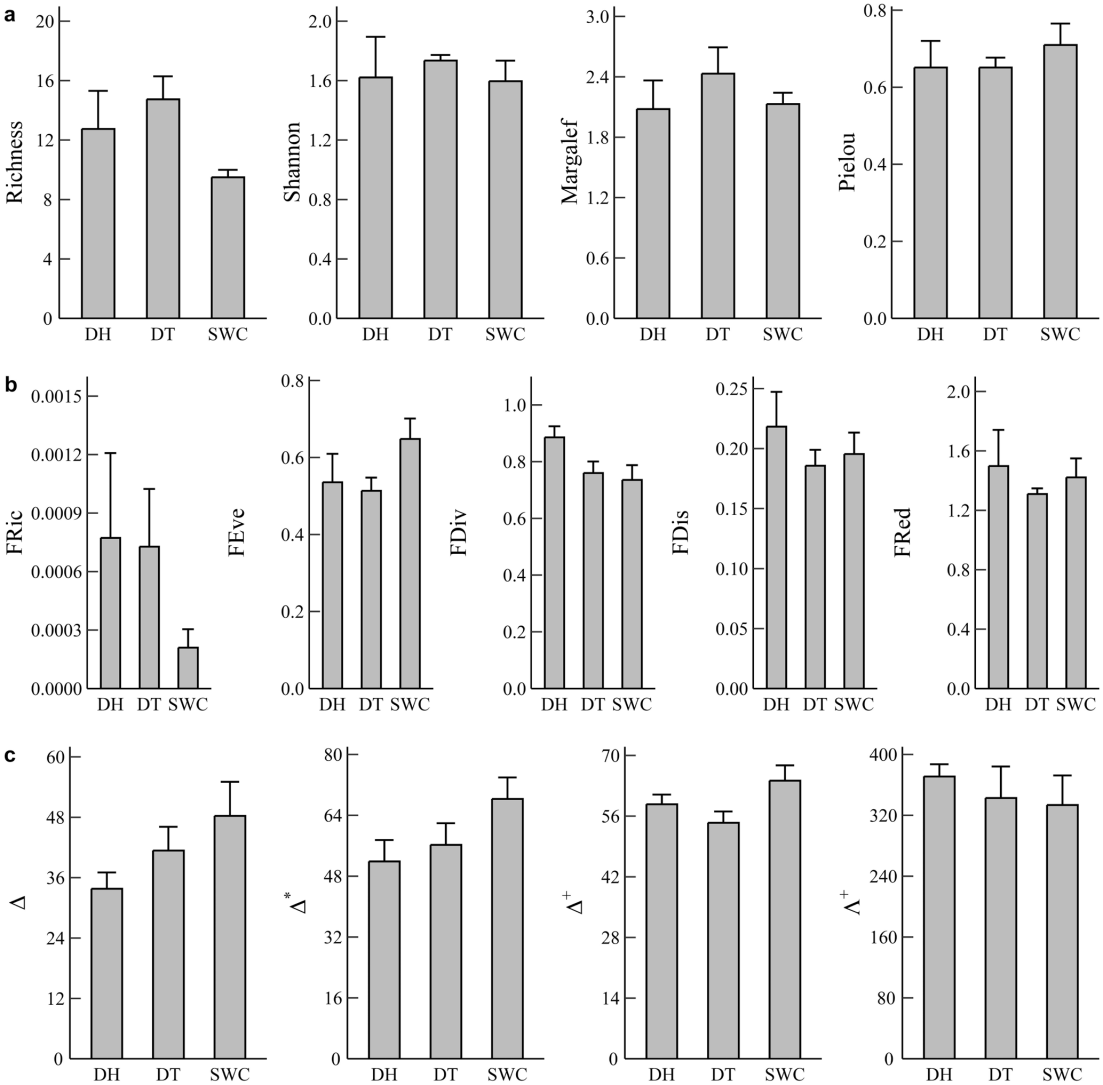
Differences of alpha taxonomic (a), functional (b), and phylogenetic (c) diversity of fish assemblages in Donghu (DH), Datun (DT), and Shuangwangcheng (SWC) reservoirs of the Eastern Route of the South‐to‐North Water Transfer Project. Error bars represent means ± SEM, *n* = 4. Analysis of variance (ANOVA) and multiple comparisons were conducted to examine differences.

None of the alpha diversity indices of the fish assemblages were found to be significantly different among the reservoirs (*p* > 0.05). Values of TDs of the assemblages in the reservoirs were low (Figure 6). Multiple linear regression showed that environmental factors had significant effects on FDs and PDs, but did not affect TDs. Effects of environmental factors on FDs were shown by ST on FDiv (*F* = 6.92, *p* < 0.05), TP on FEve (*F* = 8.89, *p* < 0.05), and pH on FRic (*F* = 5.97, *p* < 0.05), and effects on PDs were found from DIS on Δ and Δ* (*F* = 9.59, *p* < 0.05; *F* = 5.00, *p* < 0.05), NO_3_‐N on Δ and Δ* (*F* = 12.20, *p* < 0.05; *F* = 9.44, *p* < 0.05), and DIS, ST, and NO_3_‐N on Δ^+^ (DIS: *F* = 6.97, *p* < 0.05; ST: *F* = 6.72, *p* < 0.05; NO_3_‐N: *F* = 5.89, *p* < 0.05).

**FIGURE 7:**
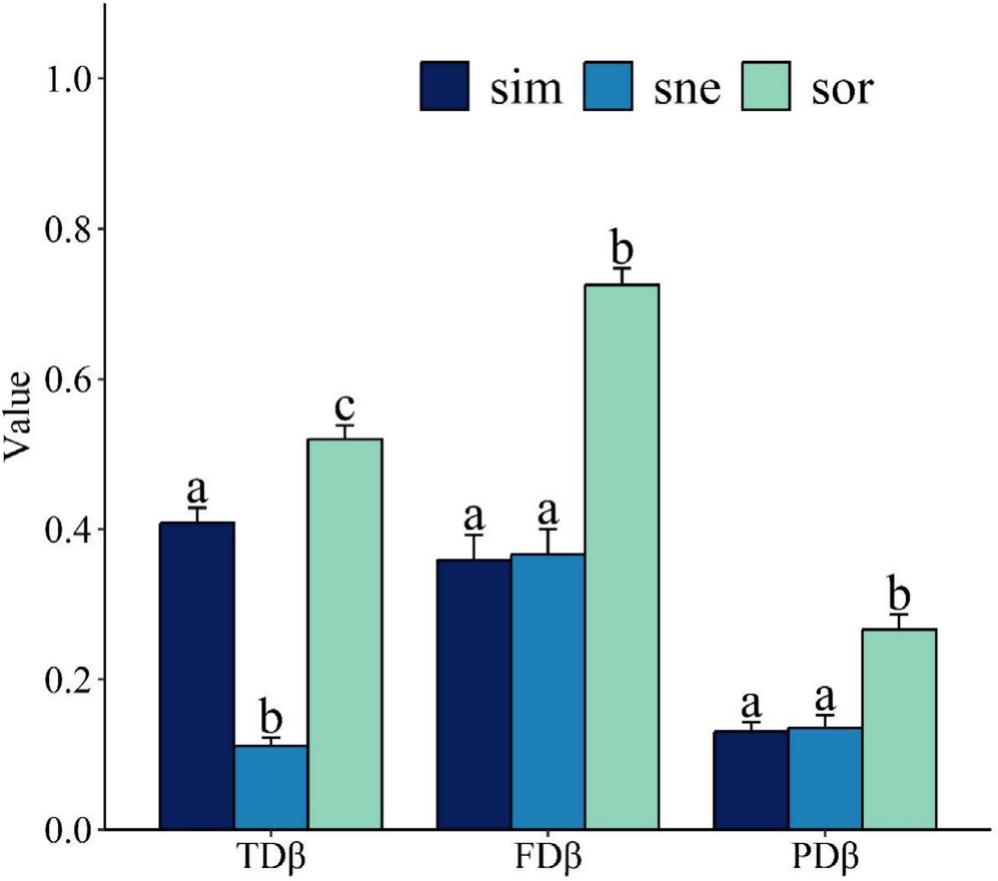
Fish taxonomic (TDβ), functional (FDβ), and phylogenetic (PDβ) beta diversity of the three reservoirs of the Eastern Route of the South‐to‐North Water Transfer Project. Bars with different lowercase letters indicate significant differences (*p* < 0.05) among groups; sim: turnover; sne: nestedness; sor: overall.

Regarding beta diversity, the variations of fish assemblages in the three reservoirs were manifested by species substitutions and nestedness in terms of functional traits and phylogeny. TDsim was significantly higher than TDsne (Kruskal–Wallis test, *p* < 0.05), whereas FDsne and PDsne accounted for more than 50% and were higher than FDsim and PDsim, respectively (Figure 7, Tables S7–S15). The variation partitioning analysis showed that environmental factors explained 46%, 32%, and 45% of the variation in TDsor, TDsim, and FDsim, respectively (Figure S3). It also revealed the significant effects of ST and water quality on TDsor and TDsim and of DIS and water quality on FDsim. The RDA results showed that NO_3_‐N and TP were the main contributors to the effects of water quality.

## 4 Discussion

In our sampling system, the distance from Dongping Lake and the first impoundment time among DH, DT, and SWC formed gradient controls along the ER‐SNWTP. Fish assemblages in the reservoirs had the same origin and entry approach; that is, from Dongping Lake. Our results showed that the composition and structure of fish assemblages varied dramatically among the reservoirs and sampling months, and no fish species were collected beyond those known to inhabit Dongping Lake. Fish assemblages were dominated by eurytopic fish in all reservoirs, and omnivorous and small‐sized fish also had dominance in species number and abundance. Distances of the water transfer, reservoir operation times, and water quality are crucial factors affecting the fish assemblages in terminal reservoirs. These results supported our hypothesis and revealed an impact mechanism of IBWTs on fish assemblages. Further research on the impact mechanisms of IBWTs on the different life history stages of fish and their relationships with ecological parameters is urgently needed. We further highlighted that the periodic disturbances of the water transfer should receive broader attention, given their substantial management implications for fish assemblage and water quality in the water bodies of IBWTs.

Our study indicates that diverted fish accompanying water transfer is likely the main cause of the impact of IBWTs on fish assemblages. Thirty‐five species, all introduced from Dongping Lake, were detected in our study, providing direct evidence of diverted fish accompanying water transfer. Although there is a consensus that IBWTs facilitate fish dispersal by breaking down natural biogeographic barriers, few studies have directly related the number of diverted fish species to water transfer (Rahel 2007; Guo et al. 2020; Liu et al. 2022). A total of 44 species were collected from five lakes, including Dongping Lake, along the ER‐SNWTP during 2017–2019, and 47 species were collected in Dongping Lake during 2010–2020 (Liu et al. 2022; Liu, Brosse, et al. 2023). Compared to the species collected in those studies, our results suggest that most of the fish species distributed along the ER‐SNWTP can be dispersed via water transfer and establish populations in recipient water bodies, which may be surprising in the dispersal scales of species using IBWTs. Previous studies have stressed the impact of IBWTs through the introduction of non‐native species, changes in water quality and hydrological regimes, and habitat modifications (Bunn and Arthington 2002; Guo et al. 2020; Yan, Chen, et al. 2023). Our results suggest that fish diverted directly by water transfer are likely to be undervalued in regard to the impact of IBWTs on fish assemblages.

Our results revealed that all dominant species have a higher dispersal chance of being diverted by the water transfer. Fish assemblages were dominated by eurytopic fish; omnivorous, small‐sized, and spawning adhesive eggs species were also well represented in the terminal reservoirs. Schmidt et al. (2020) proposed that species with specific ecological characteristics (i.e., smaller body size, high adult or larval abundance, and migratory, generalist, and pelagic habitat preferences) have higher dispersal potential, which supports our findings. Eurytopic and omnivorous species with a wider niche breadth may be more successful in the dispersal and establishment of populations in habitats with high dissimilarity among basins (Gallardo and Aldridge 2018; van Treeck et al. 2020). Small‐sized fishes can actively and/or passively enter terminal reservoirs accompanied by water transfer, owing to their easy carrying (Verhille et al. 2014; Grabowska and Przybylski 2015). Fish with spawning adhesive eggs usually have a long spawning period and a partial overlap between their peak larval abundances and periods of water transfer, which implies a high abundance of eggs and/or larvae, facilitating passive dispersal during the transfer period (Lechner et al. 2016). These species with high colonization potential and strong dispersal abilities are also pioneer species in other IBWTs worldwide. For example, *Anchoviella vaillanti* (small‐sized) and *Moenkhausia costae* (small‐sized, spawning adhesive eggs) were among the first to establish populations in the 12 recipient reservoirs along the Eastern Axis of the São Francisco River Integration Project, Brazil (Silva et al. 2023). In our study, some species with migratory behavior and pelagic habitat preferences, were not dominant in the reservoirs, which is inconsistent with the predictions of Schmidt et al. (2020). This inconsistency can be explained by different chances during the entry process due to the impact of transfer distances on different guilds of fish, and/or during the assembly process due to adapting to abiotic conditions and achieving interspecific resource differentiation (Gallardo and Aldridge 2018; Harrison et al. 2019).

The transfer distance is an important factor in shaping the diversity patterns and assemblage structures of fish in these terminal reservoirs. Significant effects of the transfer distance were detected for diversity patterns by phylogenetic alpha diversity (Δ, Δ*, Δ^+^) and functional beta diversity (FDsim), partly because phylogenetic diversity could be more sensitive to changes in the environment even at lower disturbance levels (Hall and Greenstreet 1998; Srivastava et al. 2012; Liu, Qu, et al. 2023). Furthermore, the transfer distance from Dongping Lake was the main controlling factor between SWC and DT in our research model. Our study found significant differences in assemblage structures between SWC and DT, which were identified by variations in the composition and abundance of small‐sized fish. Small‐sized fishes were detected to a lesser extent from the assemblage in SWC with the longest distance of water transfer, and the six small‐sized fishes contributed to half of the differences in assemblage structures between SWC and DT. Among the six species, four were not collected or were not dominant in SWC, and the other two species ( *Rhinogobius giurinus* and *T. bifasciatus* ) had a higher dominance in DT. These results indicate that the transfer distance likely affected the entry process of small‐sized fish, thereby shaping the diversity patterns and resulting in distinct fish assemblage structures in these terminal reservoirs along the ER–SNWTP.

The water transfer distance may affect cumulative mortality of small‐sized fish (including eggs and larvae) during the transport process, which may result in substantial differences in the impacts of IBWTs on fish assemblages in the three terminal reservoirs. The six small‐sized fishes, mainly contributors to the differences in assemblage structures between SWC and DT, have spawning peaks from March to July, which overlaps significantly with the periods of ER‐SNWTP water transfer, that is, from November to May (Wang et al. 2014; Dong et al. 2019; Qin et al. 2023). These overlaps imply that high abundances of small‐sized fish eggs and larvae were diverted and entered the terminal reservoirs via water transfer. During the early life history of fish, individuals generally experience high mortality rates because of their vulnerability to environmental factors (Hale 1999; Hitt et al. 2020). Therefore, it is reasonable to infer that the cumulative mortality of eggs and larvae during the transport process dramatically increases with increasing transfer distance. In addition, individual injuries during passage through pumping stations may be a crucial factor in the effects of transfer distance, which can lead to varied mortality rates during the transfer process for small fish, including eggs and larvae (Pracheil et al. 2016; Harrison et al. 2019; Navarro et al. 2019).

Our results support the second hypothesis that the fish periodically diverted by the water transfer may serve as a key factor in shaping fish assemblages in the water bodies of IBWTs. The operation time of the reservoirs was the main controlling factor for the periodic water transfer between DH and DT in our research model. Dramatic variations in fish composition, NPUE, and BPUE were detected among the sampling months in all reservoirs, especially for the different compositions of the dominant species. These results indicate that the fish assemblages in all of the terminal reservoirs are extremely unstable and are affected by annually diverted fish accompanied by periodic water transfer. Predictable fish assemblage succession has been demonstrated in natural lakes and artificial ecosystems (such as gravel‐pit lakes and impounded reservoirs) by many studies (Mehner et al. 2005; Agostinho et al. 2016; Martinsen et al. 2023). Fish composition may rapidly develop into a relatively stable period ranging from weeks to a few years in new lakes (Degani et al. 1998; Baber et al. 2002; Kristensen et al. 2020). Fish assemblages undergo directional development from unstable to stable phases, dominated by different fish species during reservoir aging (Kubečka 1993; Říha et al. 2009; Burgad et al. 2023). However, no significant difference in fish assemblages was found in DH compared to the other two reservoirs, though we detected the effects of reservoir operation times on assemblage succession indicated by the significant correlations with alpha diversity (FDiv, Δ^+^) and beta diversity (TDsor, TDsim). Constant pulse interferences such as annual water transfer of IBWTs can result in abrupt changes in fish assemblage and cause assemblages to directionally change over time (Erős et al. 2015; Burgad et al. 2023). Diverted fish enter reservoirs annually accompanied by the water transfers of the ER‐SNWTP, which may result in unstable fish assemblages in terminal reservoirs (Dong et al. 2019; Liu et al. 2022; Zhang et al. 2022).

Our results suggest that large volumes of eurytopic and omnivorous fishes diverted by water transfers enter the reservoirs, and they have a stronger competitive advantage, allowing them to dominate the assemblages under the periodic disturbance of water transfers. When diverted fish enter an area, their ability to assemble into assemblages depends on both abiotic variables and biotic interactions between and within the species (Leibold et al. 2004; Lucas and Baras 2008; Martinsen et al. 2023). The reservoirs in this study were built through artificial excavation and have limited primary productivity, and may only support species with specific functional traits (Nekola and White 1999; Luo et al. 2021; Li et al. 2023). Water quality, especially low nutrient loadings NO_3_‐N and TP, showed significant effects on the alpha diversity (FEve, Δ, Δ*, Δ^+^) and beta diversity (TDsor, TDsim, and FDsim), which also indicated poorly available resources in the reservoirs and competitive advantages of fishes with a wider niche breadth (Lemmens et al. 2018; Martinsen et al. 2023). The higher nestedness component of the functional and phylogenetic beta diversity indices in our results indicates the loss of closely related species with similar functional traits (Liu, Qu, et al. 2023). Eurytopic and omnivorous fish are typical species with a wider niche breadth, and have a higher potential to adapt to abiotic conditions and to achieve interspecific resource differentiation in these infertile reservoirs (Gallardo and Aldridge 2018; van Treeck et al. 2020).

Furthermore, the fish assemblage in DH had a composition and structure similar to that of DT, but differed roughly from that of SWC, when DH first impounded and received diverting fish accompanied by water transfer. Fish assemblages can develop rapidly in new water bodies where fish have convenient conditions for entry, which is consistent with our results in DH (Martinsen et al. 2023). These results indicate that the impacts of water transfer on fish assemblages may not linearly accumulate with increasing transfer numbers and that the succession of fish assemblages may be periodically disrupted in terminal reservoirs due to annual water transfer. Previous studies concerning ecological impacts associated with IBWTs focused on facilitating dispersal, changing environment, and reconfiguring processes of IBWTs (Crook et al. 2015; Havel et al. 2015; Guo et al. 2020). Our study highlights that the neglected periodic interference of annual water transfer may be a crucial factor regulating the impact of IBWTs on fish assemblages.

The assemblage succession in the terminal reservoirs may differ entirely from that in natural ones. Our results suggest that fish assemblages along the waters of IBWTs may remain in an unstable state rather than reaching a natural equilibrium due to the periodic diversion of fish by the water transfers. From an ecological perspective, fish assemblages in the waters of IBWTs, particularly in terminal reservoirs, should not be managed or utilized based on paradigms derived from natural systems (e.g., attempting to restore the original diversity or regulating fishing). Instead, management strategies should focus on maintaining ecological functions that support the objectives of IBWTs. In practice, this involves optimizing water quality regulation through rational stocking and harvesting of biomanipulation species (such as *A. nobilis* and *Hypophthalmichthys molitrix* ), thereby enhancing their role in sustaining water quality and ecosystem stability under artificial hydrological regimes. Meanwhile, water transfer distance and reservoir operation time were identified as key factors of IBWTs affecting fish assemblages in the three terminal reservoirs. Therefore, management strategies for fish assemblages should be tailored according to these factors. For example, the newly impounded DH does not require the removal of *C. carpio* and *C. auratus* (as their feeding activities disturb sediment), while DT with a longer operation time needs renewal of *A. nobilis* and *H. molitrix* populations. Additionally, SWC, characterized by both longer distance and operation time, requires targeted removal of *C. carpio* and *C. auratus* , coupled with increased stocking of *A. nobilis* and *H. molitrix* .

Our study has some limitations, including the lack of spatial extent and the use of only annual data from fish assemblage surveys. The fish assemblages of the terminal reservoirs along the ER‐SNWTP were closely related to those of Dongping Lake. However, we did not obtain permission to survey that lake. The lack of a fish survey in Dongping Lake may have partially precluded our ability to fully evaluate the impact of the ER‐SNWTP caused by fish diverted through water transfer. Detailed data on the fish assemblages in Dongping Lake were collected by Liu, Brosse, et al. (2023), providing a robust basis for our key conclusions. We detected dramatic variations in fish assemblages among the sampling months and related this instability to the impact of the ER‐SNWTP. However, existing sampling methods may miss pelagic or rare species, and our study was based on data from only one year. Long‐term data collected by a more comprehensive sampling method are advantageous for excluding natural fluctuations in fish assemblages to fully clarify the impacts of IBWTs. Our study did not differentiate among the life history stages of fish in the terminal reservoirs, which is important for understanding the impact mechanisms of IBWTs on different life stages of fish and their relationship with ecological parameters. Furthermore, eurytopic and omnivorous species dominate fish assemblages in terminal reservoirs. Research on the interspecific relationships and resource competition between eurytopic and omnivorous species in reservoirs would provide a better understanding of the impact mechanisms of IBWTs on fish assemblages. Simultaneously, examining the long‐term interactions among fish species in the terminal reservoirs requires data from extended time series. Our study lacks spatial replicates because the three terminal reservoirs we investigated are relatively unique within the context of China's IBWTs which may constrain a more accurate understanding of the ecological impacts of periodic water transfer.

## 5 Conclusion and Prospect

This study provides a theoretical basis for a deeper understanding of the ecological impacts of IBWTs on waters along diversion paths and in recipient regions. Our study revealed that the water transfer constructed fish assemblages in terminal reservoirs and the distance, number of times, and nutrient loadings of diverted water were crucial factors influencing the effects of IBWTs, indicating an impact mechanism of IBWTs on fish assemblages. The periodic interference of annual water transfer can partly explain the impact of IBWTs on fish assemblages and assemblage succession in terminal reservoirs. In our results, the dispersal scales of species utilizing IBWTs are surprising. Our results suggest the need to mitigate the impact of IBWTs on fish assemblages by avoiding overlap with the spawning peaks of small‐sized fishes when scheming transfer programs. Besides, biomanipulation can be implemented to safeguard and improve reservoir water quality based on timely fish assemblage surveys conducted after each annual water transfer period.

Global climate change is bringing great uncertainty to water resource distribution and aggravating regional imbalances, which has promoted the implementation of more IBWTs for a sustainable water supply (Yan, Lin, et al. 2023). Further efforts are required to systematically evaluate and predict the ecological impacts of IBWTs. For future work, regular fish assemblage surveys should be conducted in the water‐receiving area of the ER‐SNWTP, including Dongping Lake, with a focus on detailed analyses of fish biology and ecological niche partitioning. Meanwhile, similar water transfer systems worldwide should be examined for comparative analysis, thereby strengthening the conclusions of this study.

## Author Contributions

**Zhenhao Cheng:** formal analysis (lead), investigation (lead), methodology (lead), writing – original draft (lead), writing – review and editing (lead). **Anxiang Wang:** investigation (equal). **Yujing Cui:** funding acquisition (equal). **Lei Gao:** methodology (equal), writing – review and editing (equal). **Fei Cheng:** conceptualization (lead), writing – original draft (lead), writing – review and editing (lead). **Fengyue Shu:** conceptualization (equal), writing – review and editing (equal). **Songguang Xie:** conceptualization (equal), writing – review and editing (equal).

## Ethics Statement

During our research, we had adhered to all the necessary guidelines and protocols for the ethical use of animals in research, the legal requirements in our country, and our institutional guidelines.

## Conflicts of Interest

The authors declare no conflicts of interest.

## Supporting information


**Appendix S1:** ece372459‐sup‐0001‐AppendixS1.docx.

## Data Availability

All the required data are uploaded as Supporting Information.

## References

[ece372459-bib-0001] Agostinho, A. A. , L. C. Gomes , N. C. L. Santos , J. C. G. Ortega , and F. M. Pelicice . 2016. “Fish Assemblages in Neotropical Reservoirs: Colonization Patterns, Impacts and Management.” Fisheries Research 173: 26–36. 10.1016/j.fishres.2015.04.006.

[ece372459-bib-0002] Agostinho, A. A. , F. M. Pelicice , and L. C. Gomes . 2008. “Dams and the Fish Fauna of the Neotropical Region: Impacts and Management Related to Diversity and Fisheries.” Brazilian Journal of Biology 68: 1119–1132. 10.1590/s1519-69842008000500019. 19197482

[ece372459-bib-0003] Baber, M. J. , D. L. Childers , K. J. Babbitt , and D. H. Anderson . 2002. “Controls on Fish Distribution and Abundance in Temporary Wetlands.” Canadian Journal of Fisheries and Aquatic Sciences 59: 1441–1450. 10.1139/f02-116.

[ece372459-bib-0004] Baselga, A. 2010. “Partitioning the Turnover and Nestedness Components of Beta Diversity.” Global Ecology and Biogeography 19: 134–143. 10.1111/j.1466-8238.2009.00490.x.

[ece372459-bib-0005] Baselga, A. , and C. D. L. Orme . 2012. “betapart: An R Package for the Study of Beta Diversity.” Methods in Ecology and Evolution 3: 808–812. 10.1111/j.2041-210X.2012.00224.x.

[ece372459-bib-0006] Bernardo, J. M. , M. Ilh'eu , P. Matono , and A. M. Costa . 2003. “Interannual Variation of Fish Assemblage Structure in a Mediterranean River: Implications of Streamflow on the Dominance of Native or Exotic Species.” River Research and Applications 19: 521–532. 10.1002/rra.726.

[ece372459-bib-0007] Blanchet, F. G. , P. Legendre , and D. Borcard . 2008. “Forward Selection of Explanatory Variables.” Ecology 89: 2623–2632. 10.1890/07-0986.1. 18831183

[ece372459-bib-0008] Brucet, S. , S. Pédron , T. Mehner , et al. 2013. “Fish Diversity in European Lakes: Geographical Factors Dominate Over Anthropogenic Pressures.” Freshwater Biology 58: 1779–1793. 10.1111/fwb.12167.

[ece372459-bib-0009] Bunn, S. E. , and A. H. Arthington . 2002. “Basic Principles and Ecological Consequences of Altered Flow Regimes for Aquatic Biodiversity.” Environmental Management 30: 492–507. 10.1007/s00267-002-2737-0. 12481916

[ece372459-bib-0010] Burgad, A. A. , B. R. Kesner , and P. C. Marsh . 2023. “Long‐Term Patterns of Fish Community Structure and Decline in Native Species in a Colorado River Reservoir, Arizona and Nevada.” Transactions of the American Fisheries Society 152: 760–771. 10.1002/tafs.10437.

[ece372459-bib-0011] Chang, J. , D. L. Rabosky , S. A. Smith , and M. E. Alfaro . 2019. “An r Package and Online Resource for Macroevolutionary Studies Using the Ray‐Finned Fish Tree of Life.” Methods in Ecology and Evolution 10: 1118–1124. 10.1111/2041-210X.13182.

[ece372459-bib-0012] Cheimonopoulou, M. T. , D. C. Bobori , I. Theocharopoulos , and M. Lazaridou . 2011. “Assessing Ecological Water Quality With Macroinvertebrates and Fish: A Case Study From a Small Mediterranean River.” Environmental Management 47: 279–290. 10.1007/s00267-010-9598-8. 21170710

[ece372459-bib-0013] Chen, Y. 1998. Fauna Sinica China. Science Press.

[ece372459-bib-0014] Cheng, Q. T. , and C. W. Zhou . 1997. The Fishes of Shandong Province. Shandong Science and Technology Press.

[ece372459-bib-0015] Clarke, K. R. 1993. “Non‐Parametric Multivariate Analyses of Changes in Community Structure.” Australian Journal of Ecology 18: 117–143. 10.1111/j.1442-9993.1993.tb00438.x.

[ece372459-bib-0016] Clarke, K. R. , and R. N. Gorley . 2006. PRIMER v6: User Manual/Tutorial. PRIMER‐E.

[ece372459-bib-0017] Clarke, K. R. , and R. M. Warwick . 1998. “A Taxonomic Distinctness Index and Its Statistical Properties.” Journal of Applied Ecology 35: 523–531. 10.1046/j.1365-2664.1998.3540523.x.

[ece372459-bib-0018] Clarke, K. R. , and R. M. Warwick . 2001. “A Further Biodiversity Index Applicable to Species Lists: Variation in Taxonomic Distinctness.” Marine Ecology Progress Series 216: 265–278.

[ece372459-bib-0019] Crook, D. A. , W. H. Lowe , F. W. Allendorf , et al. 2015. “Human Effects on Ecological Connectivity in Aquatic Ecosystems: Integrating Scientific Approaches to Support Management and Mitigation.” Science of the Total Environment 534: 52–64. 10.1016/j.scitotenv.2015.04.034. 25917446

[ece372459-bib-0020] Daga, V. S. , V. M. Azevedo‐Santos , F. M. Pelicice , et al. 2020. “Water Diversion in Brazil Threatens Biodiversity.” Ambio 49: 165–172. 10.1007/s13280-019-01189-8. 31030418 PMC6888777

[ece372459-bib-0021] Degani, G. , Y. Yehuda , J. Jackson , and M. Gophen . 1998. “Temporal Variation in Fish Community Structure in a Newly Created Wetland Lake (Lake Agmon) in Israel.” Wetlands Ecology and Management 6: 151–157. 10.1023/a:1008463918715.

[ece372459-bib-0022] Dević, G. 2015. “Environmental Impacts of Reservoirs.” In Environmental Indicators, edited by R. H. Armon and O. Hänninen , 561–575. Springer Netherlands. 10.1007/978-94-017-9499-2_33.

[ece372459-bib-0023] Dobbs, G. R. , N. Liu , P. V. Caldwell , et al. 2023. “Inter‐Basin Surface Water Transfers Database for Public Water Supplies in Conterminous United States, 1986–2015.” Scientific Data 10: 255. 10.1038/s41597-023-02148-5. 37149676 PMC10164180

[ece372459-bib-0024] Dong, X. , T. Xiang , T. Ju , et al. 2019. “Age, Growth, Mortality and Recruitment of Thin Sharpbelly *Toxabramis swinhonis* Günther, 1873 in Three Shallow Lakes Along the Middle and Lower Reaches of the Yangtze River Basin, China.” PeerJ 7: e6772. 10.7717/peerj.6772. 31011492 PMC6464026

[ece372459-bib-0025] Erős, T. , P. Takács , I. Czeglédi , P. Sály , and A. Specziár . 2015. “Taxonomic‐ and Trait‐Based Recolonization Dynamics of a Riverine Fish Assemblage Following a Large‐Scale Human‐Mediated Disturbance: The Red Mud Disaster in Hungary.” Hydrobiologia 758: 31–45. 10.1007/s10750-015-2262-9.

[ece372459-bib-0026] Feng, K. , W. Deng , H. Li , et al. 2023. “Direct and Indirect Effects of a Fishing Ban on Lacustrine Fish Community Do Not Result in a Full Recovery.” Journal of Applied Ecology 60: 2210–2222. 10.1111/1365-2664.14491.

[ece372459-bib-0027] Feng, T. , C. Wang , J. Hou , et al. 2018. “Effect of Inter‐Basin Water Transfer on Water Quality in an Urban Lake: A Combined Water Quality Index Algorithm and Biophysical Modelling Approach.” Ecological Indicators 92: 61–71. 10.1016/j.ecolind.2017.06.044.

[ece372459-bib-0028] Gallardo, B. , and D. C. Aldridge . 2018. “Inter‐Basin Water Transfers and the Expansion of Aquatic Invasive Species.” Water Research 143: 282–291. 10.1016/j.watres.2018.06.056. 29986238

[ece372459-bib-0029] Ghassemi, F. , and I. R. White . 2007. Inter‐Basin Water Transfer: Case Studies From Australia, United States, Canada, China and India. Cambridge University Press.

[ece372459-bib-0030] Gibbins, C. N. , C. Soulsby , M. J. Jeffries , and R. Acornley . 2002. “Developing Ecologically Acceptable River Flow Regimes: A Case Study of Kielder Reservoir and the Kielder Water Transfer System.” Fisheries Management and Ecology 8: 463–485. 10.1046/j.1365-2400.2001.00274.x.

[ece372459-bib-0031] Grabowska, J. , and M. Przybylski . 2015. “Life‐History Traits of Non‐Native Freshwater Fish Invaders Differentiate Them From Natives in the Central European Bioregion.” Reviews in Fish Biology and Fisheries 25: 165–178. 10.1007/s11160-014-9375-5.

[ece372459-bib-0032] Grant, E. H. C. , H. J. Lynch , R. Muneepeerakul , M. Arunachalam , I. Rodríguez‐Iturbe , and W. F. Fagan . 2012. “Interbasin Water Transfer, Riverine Connectivity, and Spatial Controls on Fish Biodiversity.” PLoS One 7: e34170. 10.1371/journal.pone.0034170. 22470533 PMC3314595

[ece372459-bib-0033] Guo, C. , Y. Chen , R. E. Gozlan , et al. 2020. “Patterns of Fish Communities and Water Quality in Impounded Lakes of China's South‐to‐North Water Diversion Project.” Science of the Total Environment 713: 136515. 10.1016/j.scitotenv.2020.136515. 31951840

[ece372459-bib-0034] Guo, C. , Y. Chen , H. Liu , et al. 2019. “Modelling Fish Communities in Relation to Water Quality in the Impounded Lakes of China's South‐to‐North Water Diversion Project.” Ecological Modelling 397: 25–35. 10.1016/j.ecolmodel.2019.01.014.

[ece372459-bib-0035] Guo, X. , T. Hu , T. Zhang , and Y. Lv . 2012. “Bilevel Model for Multi‐Reservoir Operating Policy in Inter‐Basin Water Transfer‐Supply Project.” Journal of Hydrology 424‐425: 252–263. 10.1016/j.jhydrol.2012.01.006.

[ece372459-bib-0036] Hale, M. E. 1999. “Locomotor Mechanics During Early Life History: Effects of Size and Ontogeny on Fast‐Start Performance of Salmonid Fishes.” Journal of Experimental Biology 202: 1465–1479. 10.1242/jeb.202.11.1465. 10229693

[ece372459-bib-0037] Hall, S. J. , and S. P. Greenstreet . 1998. “Taxonomic Distinctness and Diversity Measures: Responses in Marine Fish Communities.” Marine Ecology Progress Series 166: 227–229.

[ece372459-bib-0038] Harrison, P. M. , E. G. Martins , D. A. Algera , et al. 2019. “Turbine Entrainment and Passage of Potadromous Fish Through Hydropower Dams: Developing Conceptual Frameworks and Metrics for Moving Beyond Turbine Passage Mortality.” Fish and Fisheries 20: 403–418. 10.1111/faf.12349.

[ece372459-bib-0039] Havel, J. E. , K. E. Kovalenko , S. M. Thomaz , S. Amalfitano , and L. B. Kats . 2015. “Aquatic Invasive Species: Challenges for the Future.” Hydrobiologia 750: 147–170. 10.1007/s10750-014-2166-0. 32214452 PMC7087615

[ece372459-bib-0040] Hinton, M. , and M. Maunder . 2004. “Methods for Standardizing CPUE and How to Select Among Them.” ICCAT Collective Volume of Scientific Papers 56, no. 1: 169–177.

[ece372459-bib-0041] Hitt, N. P. , K. M. Rogers , Z. A. Kelly , J. Henesy , and J. E. Mullican . 2020. “Fish Life History Trends Indicate Increasing Flow Stochasticity in an Unregulated River.” Ecosphere 11: e03026. 10.1002/ecs2.3026.

[ece372459-bib-0042] Jia, Y. , Y. Jiang , Y. Liu , et al. 2021. “Unravelling Fish Community Assembly in Shallow Lakes: Insights From Functional and Phylogenetic Diversity.” Reviews in Fish Biology and Fisheries 32: 623–644. 10.1007/s11160-021-09688-2.

[ece372459-bib-0043] Jurasinski, G. , V. Retzer , and C. Beierkuhnlein . 2009. “Inventory, Differentiation, and Proportional Diversity: A Consistent Terminology for Quantifying Species Diversity.” Oecologia 159: 15–26. 10.1007/s00442-008-1190-z. 18953572

[ece372459-bib-0044] Kristensen, E. , K. Sand‐Jensen , J. S. B. Kristensen , M. E. Pedersen , L. Baastrup‐Spohr , and T. Kragh . 2020. “Early Fish Colonization and Community Development in a Shallow Re‐Established Lake.” Ecological Engineering 155: 105956. 10.1016/j.ecoleng.2020.105956.

[ece372459-bib-0045] Kubečka, J. 1993. “Succession of Fish Communities in Reservoirs of Central and Eastern Europe.” In Comparative Reservoir Limnology and Water Quality Management, 153–168. Springer Netherlands. 10.1007/978-94-017-1096-1_11.

[ece372459-bib-0046] Laliberté, E. , P. Legendre , and B. Shipley . 2015. “FD: Measuring Functional Diversity (FD) From Multiple Traits, and Other Tools for Functional Ecology.” Package 1.0–12. https://CRAN.R‐project.org/package=FD. 10.1890/08-2244.1 20380219

[ece372459-bib-0047] Lechner, A. , H. Keckeis , and P. Humphries . 2016. “Patterns and Processes in the Drift of Early Developmental Stages of Fish in Rivers: A Review.” Reviews in Fish Biology and Fisheries 26: 471–489. 10.1007/s11160-016-9437-y.

[ece372459-bib-0048] Legendre, P. , and L. Legendre . 2012. Numerical Ecology (3rd English ed.). Elsevier.

[ece372459-bib-0049] Leibold, M. A. , M. Holyoak , N. Mouquet , et al. 2004. “The Metacommunity Concept: A Framework for Multi‐Scale Community Ecology.” Ecology Letters 7: 601–613. 10.1111/j.1461-0248.2004.00608.x.

[ece372459-bib-0050] Lemmens, P. , S. A. J. Declerck , K. Tuytens , M. Vanderstukken , and L. De Meester . 2018. “Bottom‐Up Effects on Biomass Versus Top‐Down Effects on Identity: A Multiple‐Lake Fish Community Manipulation Experiment.” Ecosystems 21: 166–177. 10.1007/s10021-017-0144-x.

[ece372459-bib-0051] Li, Y. , K. Ma , W. Song , et al. 2023. “Environmental Heterogeneity and Dispersal Limitation Simultaneously Determine the Spatial Scaling of Different Microbial Functional Groups.” Science of the Total Environment 885: 163854. 10.1016/j.scitotenv.2023.163854. 37142009

[ece372459-bib-0052] Liu, H. , S. Brosse , X. Qu , W. Xia , X. Li , and Y. Chen . 2023. “Land Use Outweighs Other Stressors in Declining Fish Biodiversity in Lakes of Eastern China During the 1980s‐2010s.” Ecological Indicators 152: 110390. 10.1016/j.ecolind.2023.110390.

[ece372459-bib-0053] Liu, H. , Y. Chen , R. Gozlan , et al. 2022. “Fish Diversity Reduction and Assemblage Structure Homogenization in Lakes: A Case Study on Unselective Fishing in China.” Water Biology and Security 1: 100055. 10.1016/j.watbs.2022.100055.

[ece372459-bib-0054] Liu, H. , X. Qu , W. Xia , and Y. Chen . 2023. “Taxonomic, Functional, and Phylogenetic Diversity Patterns Reveal Different Processes Shaping River Fish Assemblages in the Eastern Huai River Basin, China.” Water Biology and Security 2: 100078. 10.1016/j.watbs.2022.100078.

[ece372459-bib-0055] Lucas, M. C. , and E. Baras . 2008. Migration of Freshwater Fishes. Blackwell Science.

[ece372459-bib-0056] Luo, J. , G. Huang , Y. Shao , J. Liu , and Q. Xie . 2021. “Laboratory Test and Numerical Simulation of Composite Geomembrane Leakage in Plain Reservoir.” Open Geosciences 13: 651–662. 10.1515/geo-2020-0247.

[ece372459-bib-0057] Ma, Y. , J. Chang , A. Guo , L. Wu , J. Yang , and L. Chen . 2020. “Optimizing Inter‐Basin Water Transfers From Multiple Sources Among Interconnected River Basins.” Journal of Hydrology 590: 125461. 10.1016/j.jhydrol.2020.125461.

[ece372459-bib-0058] Martinsen, K. T. , E. Kristensen , L. Baastrup‐Spohr , et al. 2023. “Environmental Predictors of Lake Fish Diversity Across Gradients in Lake Age and Spatial Scale.” Freshwater Biology 68: 1122–1135. 10.1111/fwb.14090.

[ece372459-bib-0059] McKinney, M. L. , and J. L. Lockwood . 1999. “Biotic Homogenization: A Few Winners Replacing Many Losers in the Next Mass Extinction.” Trends in Ecology & Evolution 14: 450–453. 10.1016/S0169-5347(99)01679-1. 10511724

[ece372459-bib-0060] Mehner, T. , M. Diekmann , U. Brämick , and R. Lemcke . 2005. “Composition of Fish Communities in German Lakes as Related to Lake Morphology, Trophic State, Shore Structure and Human‐Use Intensity.” Freshwater Biology 50: 70–85. 10.1111/j.1365-2427.2004.01294.x.

[ece372459-bib-0061] Navarro, A. , C. A. Boys , W. Robinson , et al. 2019. “Tolerable Ranges of Fluid Shear for Early Life‐Stage Fishes: Implications for Safe Fish Passage at Hydropower and Irrigation Infrastructure.” Marine and Freshwater Research 70: 1503–1512. 10.1071/MF18131.

[ece372459-bib-0062] Nekola, J. C. , and P. S. White . 1999. “The Distance Decay of Similarity in Biogeography and Ecology.” Journal of Biogeography 26: 867–878. 10.1046/j.1365-2699.1999.00305.x.

[ece372459-bib-0063] Ni, Y. , and H. L. Wu . 2006. Fishes of Jiangsu Province. China Agriculture Press.

[ece372459-bib-0064] Oksanen, J. , F. Guillaume Blanchet , M. Friendly , et al. 2020. “vegan: Community Ecology Package.” R package version 2.5–7. https://CRAN.R‐project.org/package=vegan.

[ece372459-bib-0065] Peres‐Neto, P. , P. Legendre , S. Dray , and D. Borcard . 2006. “Variation Partitioning of Species Data Matrices: Estimation and Comparison of Fractions.” Ecology 87: 2614–2625. 10.1890/0012-9658(2006)87. 17089669

[ece372459-bib-0066] Pinkas, L. , M. S. Oliphant , and I. L. K. Iverson . 1971. “Food Habits of Albacore, Bluefin Tuna, and Bonito in Californian Waters.” Fishery Bulletin 152: 11–105.

[ece372459-bib-0067] Pracheil, B. M. , C. R. DeRolph , M. P. Schramm , and M. S. Bevelhimer . 2016. “A Fish‐Eye View of Riverine Hydropower Systems: The Current Understanding of the Biological Response to Turbine Passage.” Reviews in Fish Biology and Fisheries 26: 153–167. 10.1007/s11160-015-9416-8.

[ece372459-bib-0068] Prchalová, M. , T. Mrkvička , J. Peterka , M. Čech , L. Berec , and J. Kubečka . 2011. “A Model of Gillnet Catch in Relation to the Catchable Biomass, Saturation, Soak Time and Sampling Period.” Fisheries Research 107: 201–209. 10.1016/j.fishres.2010.10.021.

[ece372459-bib-0069] Purvis, L. , and A. Dinar . 2020. “Are Intra‐ and Inter‐Basin Water Transfers a Sustainable Policy Intervention for Addressing Water Scarcity?” Water Security 9: 100058. 10.1016/j.wasec.2019.100058.

[ece372459-bib-0070] Qin, J. , B. V. Schmidt , L. Zhang , F. Cheng , and S. Xie . 2023. “Water Transfer Determines the Regional Spread Dynamics of Non‐Native Fish Species.” Water Biology and Security 2: 100135. 10.1016/j.watbs.2023.100135.

[ece372459-bib-0071] Qin, J. , S. Xie , and F. Cheng . 2020. “Broad Diet Composition and Seasonal Feeding Variation Facilitate Successful Invasion of the Shimofuri Goby (*Tridentiger bifasciatus*) in a Water Transfer System.” Water (Basel) 12: 3411. 10.3390/w12123411.

[ece372459-bib-0072] R Development Core Team . 2020. R: A Language and Environment for Statistical Computing. R Foundation for Statistical Computing.

[ece372459-bib-0073] Rabosky, D. L. , J. Chang , P. O. Title , et al. 2018. “An Inverse Latitudinal Gradient in Speciation Rate for Marine Fishes.” Nature 559: 392–395. 10.1038/s41586-018-0273-1. 29973726

[ece372459-bib-0074] Rahel, F. J. 2007. “Biogeographic Barriers, Connectivity and Homogenization of Freshwater Faunas: It's a Small World After All.” Freshwater Biology 52: 696–710. 10.1111/j.1365-2427.2006.01708.x.

[ece372459-bib-0075] Ramos, T. P. A. , S. Y. Lustosa‐Costa , R. M. O. Lima , J. E. d. L. Barbosa , and R. F. Menezes . 2021. “First Record of *Moenkhausia costae* (Steindachner 1907) in the Paraíba Do Norte Basin After the São Francisco River Diversion.” Biota Neotropica 21: e20201049. 10.1590/1676-0611-BN-2020-1049.

[ece372459-bib-0076] Ricotta, C. , F. de Bello , M. Moretti , M. Caccianiga , B. E. L. Cerabolini , and S. Pavoine . 2016. “Measuring the Functional Redundancy of Biological Communities: A Quantitative Guide.” Methods in Ecology and Evolution 7: 1386–1395. 10.1111/2041-210X.12604.

[ece372459-bib-0077] Říha, M. , J. Kubečka , M. Vašek , et al. 2009. “Long‐Term Development of Fish Populations in the Římov Reservoir.” Fisheries Management and Ecology 16: 121–129. 10.1111/j.1365-2400.2008.00650.x.

[ece372459-bib-0078] Schmidt, B. V. , Z. Wang , P. Ren , et al. 2020. “A Review of Potential Factors Promoting Fish Movement in Inter‐Basin Water Transfers, With Emergent Patterns From a Trait‐Based Risk Analysis for a Large‐Scale Project in China.” Ecology of Freshwater Fish 29: 790–807. 10.1111/eff.12530.

[ece372459-bib-0079] Schweiger, O. , S. Klotz , W. Durka , and I. Kühn . 2008. “A Comparative Test of Phylogenetic Diversity Indices.” Oecologia 157: 485–495. 10.1007/s00442-008-1082-2. 18566837

[ece372459-bib-0080] Silva, A. L. B. , G. A. Galvão , A. A. F. Rocha , et al. 2023. “Ichthyofauna on the Move: Fish Colonization and Spread Through the São Francisco Inter‐Basin Water Transfer Project.” Neotropical Ichthyology 21: e220016. 10.1590/1982-0224-2022-0016.

[ece372459-bib-0081] Silveira, R. A. , J. Ferrer , F. G. Becker , and S. M. Hartz . 2017. “Biological Invasion at an Early Stage? First Record of the Banjo Catfish *Pseudobunocephalus iheringii* (Siluriformes: Aspredinidae) in the Tramandaí River Basin, Brazil and the Potential Invasion Pathway to This System.” Brazilian Journal of Biology 77: 890–892. 10.1590/1519-6984.01716. 28355386

[ece372459-bib-0082] Srivastava, D. S. , M. W. Cadotte , A. A. M. MacDonald , R. G. Marushia , N. Mirotchnick , and A. Mooers . 2012. “Phylogenetic Diversity and the Functioning of Ecosystems.” Ecology Letters 15: 637–648. 10.1111/j.1461-0248.2012.01795.x. 22583836

[ece372459-bib-0083] Sun, S. , X. Zhou , H. Liu , et al. 2021. “Unraveling the Effect of Inter‐Basin Water Transfer on Reducing Water Scarcity and Its Inequality in China.” Water Research 194: 116931. 10.1016/j.watres.2021.116931. 33636664

[ece372459-bib-0084] Swift, C. C. , S. Howard , J. Mulder , D. J. Pondella II , and T. P. Keegan . 2014. “Expansion of the Non‐Native Mississippi Silverside, *Menidia audens* (Pisces, Atherinopsidae), Into Fresh and Marine Waters of Coastal Southern California.” Bulletin of the Southern California Academy of Sciences 113: 153–164. 10.3160/0038-3872-113.3.153.

[ece372459-bib-0085] The State Council of the People's Republic of China . 2022. “China's Mega Water Diversion Project Benefits 150 mln People.” https://www.gov.cn/xinwen/2022‐08/25/content_5706843.htm.

[ece372459-bib-0086] van Treeck, R. , J. Van Wichelen , and C. Wolter . 2020. “Fish Species Sensitivity Classification for Environmental Impact Assessment, Conservation and Restoration Planning.” Science of the Total Environment 708: 135173. 10.1016/j.scitotenv.2019.135173. 31796278

[ece372459-bib-0087] Verhille, C. E. , J. B. Poletto , D. E. Cocherell , et al. 2014. “Larval Green and White Sturgeon Swimming Performance in Relation to Water‐Diversion Flows.” Conservation Physiology 2: 1–14. 10.1093/conphys/cou031. PMC4806727 27293652

[ece372459-bib-0088] Villéger, S. , S. Brosse , M. Mouchet , D. Mouillot , and M. J. Vanni . 2017. “Functional Ecology of Fish: Current Approaches and Future Challenges.” Aquatic Sciences 79: 783–801. 10.1007/s00027-017-0546-z.

[ece372459-bib-0089] Villéger, S. , N. W. H. Mason , and D. Mouillot . 2008. “New Multidimensional Functional Diversity Indices for a Multifaceted Framework in Functional Ecology.” Ecology 89: 2290–2301. 10.1890/07-1206.1. 18724739

[ece372459-bib-0090] Vitule, J. R. , V. M. Azevedo‐Santos , V. Salete Daga , et al. 2015. “Brazil's Drought: Protect Biodiversity.” Science 347: 1427–1428. 10.1126/science.347.6229.1427-b. 25814574

[ece372459-bib-0091] Wang, T. , D. Huang , J. Shen , Y. Chen , G. Sun , and H. Wang . 2014. “Batch Fecundity and Spawning Frequency of Invasive *Hemiculter leucisculus* (Basilewsky, 1855) in Erhai Lake, China.” Environmental Biology of Fishes 97: 1161–1168. 10.1007/s10641-013-0205-8.

[ece372459-bib-0092] Xia, W. , B. Zhu , S. Zhang , et al. 2022. “Climate, Hydrology, and Human Disturbance Drive Long‐Term (1988–2018) Macrophyte Patterns in Water Diversion Lakes.” Journal of Environmental Management 319: 115726. 10.1016/j.jenvman.2022.115726. 35849931

[ece372459-bib-0093] Yan, H. , S. Chen , X. Liu , et al. 2023. “Investigations of Fish Assemblages Using Two Methods in Three Terminal Reservoirs of the East Route of South‐to‐North Water Transfer Project, China.” Animals 13: 1614. 10.3390/ani13101614. 37238044 PMC10215622

[ece372459-bib-0094] Yan, H. , Y. Lin , Q. Chen , et al. 2023. “A Review of the Eco‐Environmental Impacts of the South‐to‐North Water Diversion: Implications for Interbasin Water Transfers.” Engineering 30: 161–169. 10.1016/j.eng.2023.05.012.

[ece372459-bib-0095] Yan, H. , A. Wang , F. Cheng , et al. 2025. “Stochastic Assembly Process Indicates High Risks of Water Transfer on Fish Communities in Waters Along the East Route of the South‐to‐North Water Transfer Project, China.” Global Ecology and Conservation 61: e03642. 10.1016/j.gecco.2025.e03642.

[ece372459-bib-0096] Yang, L. H. , J. L. Bastow , K. O. Spence , and A. N. Wright . 2008. “What Can We Learn From Resource Pulses?” Ecology 89: 621–634. 10.1890/07-0175.1. 18459327

[ece372459-bib-0097] Zhang, Z. , N. Yu , Y. Zhang , et al. 2022. “Characteristics and Source Analysis of Water Pollution in Dry Season (November to March) of Dongping Lake (China).” Agricultural Water Management 273: 107875. 10.1016/j.agwat.2022.107875.

