# Supplementary material for: The Impact Mechanism of Inter‐Basin Water Transfer on Fish Assemblages Was Revealed Using a Model of Terminal Reservoirs
Source: Ecol Evol. 2025 Nov 2;15(11):e72459. doi: 10.1002/ece3.72459 (PMC12580297; doi:10.1002/ece3.72459)
Supplement: Supplementary file 1 — Appendix S1: ece372459‐sup‐0001‐AppendixS1.docx. [file ECE3-15-e72459-s001.docx]

**The impact mechanism of inter-basin water transfer on fish assemblages was revealed using a model of terminal reservoirs**

Zhenhao Cheng ^a^, Anxiang Wang ^b^, Yujing Cui ^c^, Lei Gao ^d^, Fei Cheng ^e *^, Fengyue Shu ^a *^, Songguang Xie ^e^

^a^ School of Life Sciences, Qufu Normal University, 57 Jingxuan West Road, Qufu, 273165, Shandong, China

^b^ Key Laboratory of Freshwater Fish Reproduction and Development (Ministry of Education), College of Fisheries, Southwest University, Chongqing, 400715, China

^c^ Huaihe River and Xiaoqinghe River Basin Water Conservancy Management and Service Center of Shandong Province, Water Resources Department of Shandong Province, Jinan, 250100, China

^d^ Yangtze River Fisheries Research Institute, Chinese Academy of Fishery Sciences, Wuhan, 430223, China

^e^ School of Marine Biology and Fisheries, Hainan University, Haikou, 570228, China

^*^ Corresponding author: chengfei@hainanu.edu.cn (F. Cheng), shfyue01@163.com (F.Y. Shu)

***Appendix***

The formulae we used to calculate the alpha taxonomic diversity are as follows:

Species richness:

$$\text{Richness}\text{=}\text{n}$$

where n is the total number of species with abundance greater than 0.

Shannon-Wiener index：

$$\text{Shannon=-}\sum_{\text{i}\text{=1}}^{\text{ S}} \text{P}_{\text{i}}\ln\text{P}_{\text{i}}$$

where Pi is the relative abundance of fish species i, and S is the number of fish species at a certain site.

Pielou’s evenness：

$$\text{Pielou}\text{=}{\text{S}\text{h}\text{annon}}/{\ln\text{S}}$$

Margalef ’s richness index：

$$\text{Margalef}\text{=}{\text{（}\text{S-1}\text{）}}/{\ln\text{N}}$$

where N is the sum of the individual number of all species.

References:

Shannon, C. E., 1948. A mathematical theory of communication. Bell System Technical Journal 27, 379-423. https://doi.org/10.1002/j.1538-7305.1948.tb01338.x.

Pielou, E. C., 1966. The measurement of diversity in different types of biological collections. Journal of Theoretical Biology 13, 131-144. https://doi.org/10.1016/0022-5193(66)90013-0.

Margalef, R., 1958. Information theory in ecology. General Systematics 3, 36-71.

Table S1 Water quality parameters (Mean ± SE) detected from the three terminal reservoirs of the Eastern Route of the South-to-North Water Transfer Project during July 2021 to May 2022

|  |  | **DH** | | | | **DT** | | | | **SWC** | | | |
| --- | --- | --- | --- | --- | --- | --- | --- | --- | --- | --- | --- | --- | --- |
| **Parameters (unit)** | Code | July | October | January | May | July | October | January | May | July | October | January | May |
| **Water temperature (°C)** | WT | 29.25±0.10 | 20.58±0.06 | 4.43±0.06 | 21.9±0.19 | 28.13±0.20 | 18.65±0.03 | 1.78±0.10 | 22.18±0.11 | 29.08±0.20 | 19.18±0.06 | 2.03±0.06 | 21.1±0.04 |
| **Dissolved oxygen (mg/l)** | DO | 7.35±0.08 | 8.66±0.07 | 10.89±0.53 | 8.95±0.12 | 7.89±0.21 | 11.54±0.31 | 12.98±0.78 | 8.72±0.10 | 7.47±0.12 | 9.50±0.08 | 12.97±0.52 | 8.33±0.16 |
| **Conductivity (µS/cm)** | Cond | 1060.00±5.87 | 835.25±0.85 | 913.00±21.76 | 926.50±3.71 | 1280.75±5.86 | 992.50±0.65 | 1130.75±4.50 | 1130.75±14.80 | 1156.75±5.34 | 874.75±1.25 | 1025.00±48.56 | 994.00±1.08 |
| **Salinity(%)** | SAL | 0.48±0.00 | 0.45±0.00 | 0.46±0.01 | 0.49±0.00 | 0.60±0.00 | 0.56±0.00 | 0.58±0.00 | 0.58±0.00 | 0.53±0.00 | 0.49±0.00 | 0.53±0.03 | 0.53±0.00 |
| **pH** | pH | 5.76±0.57 | 9.07±0.01 | 8.89±0.01 | 8.27±0.06 | 7.52±0.22 | 9.04±0.02 | 8.68±0.06 | 8.25±0.04 | 8.25±0.07 | 9.21±0.01 | 9.01±0.04 | 8.10±0.07 |
| **Seccchi depth (m)** | SD | 1.80±0.04 | 4.00±0.23 | 14.80±0.09 | 7.68±0.11 | 2.15±0.03 | 1.43±0.15 | 4.65±0.06 | 3.10±0.23 | 1.53±0.05 | 1.20±0.04 | 2.63±0.05 | 3.43±0.28 |
| **Ammonia (mg/L)** | NH_4_-N | 0.19±0.03 | 0.46±0.03 | 0.41±0.01 | 0.23±0.00 | 0.19±0.01 | 0.49±0.04 | 0.35±0.01 | 0.21±0.01 | 0.20±0.01 | 0.49±0.05 | 0.32±0.02 | 0.26±0.02 |
| **Nitrate (mg/L)** | NO_3_-N | 0.30±0.01 | 0.49±0.02 | 1.09±0.02 | 5.57±0.13 | 0.66±0.00 | 0.23±0.01 | 0.26±0.04 | 6.81±0.09 | 0.96±0.02 | 0.5±0.01 | 2.81±0.06 | 4.76±0.06 |
| **Total nitrogen (mg/L)** | TN | 0.63±0.01 | 1.06±0.08 | 1.97±0.18 | 6.4±0.20 | 1.12±0.07 | 1.09±0.07 | 1.67±0.32 | 7.61±0.55 | 1.32±0.04 | 1.58±0.33 | 3.47±0.10 | 5.68±0.08 |
| **Total phosphorus (mg/L)** | TP | 0.01±0.00 | 0.07±0.00 | 0.07±0.02 | 0.10±0.01 | 0.004±0.00 | 0.07±0.00 | 0.07±0.01 | 0.50±0.06 | 0.01±0.00 | 0.08±0.00 | 0.11±0.01 | 0.50±0.05 |
| **Chlorophyll a (μg/L)** | Chl.a | 0.89±0.03 | 0.22±0.01 | 0.12±0.03 | 0.17±0.01 | 0.43±0.10 | 0.47±0.06 | 0.08±0.01 | 0.22±0.03 | 0.83±0.09 | 0.43±0.02 | 0.29±0.02 | 0.15±0.00 |

DH: Donghu Reservoir, DT: Datun Reservoir, SWC: Shuangwangcheng Reservoir

**Methods**

Water temperature, dissolved oxygen, conductivity, salinity, and pH were detected using a portable multi-parameter water quality meter (YSI Professional Plus, Xylem, Inc., USA); seccchi depth was detected using Secchi Disc; total nitrogen, ammonia, nitrate, nitrite, total phosphorus and chlorophyll a were sampled and detected in the laboratory following APHA (2005) and Liu et al. (2023).

APHA, 2005. Standard Methods for the Examination of Water and Wastewater, twenty-first ed. American Public Health Association, American Water Works Association, Water Environment Federation, Washington, DC, USA.

Liu, H., Qu, X., Xia, W., Chen, Y., 2023. Taxonomic, functional, and phylogenetic diversity patterns reveal different processes shaping river fish assemblages in the Eastern Huai River Basin, China. Water Biology and Security 2, 100078. https://doi.org/10.1016/j.watbs.2022.100078.

Table S2 Basic characteristics of the three terminal reservoirs of the Eastern Route of the South-to-North Water Transfer Project

| **Factor** | **DH** | **DT** | **SWC** |
| --- | --- | --- | --- |
| **Year of first impoundment** | 2020 | 2013 | 2013 |
| **Distance of water transfer (km)** | 160 | 190 | 290 |
| **Mean water depth (± SE, m)** | 10.8 ± 0.2 | 7.8 ± 0.5 | 8.7 ± 0.3 |
| **Aggregate storage capacity (km^3^)** | 0.06 | 0.05 | 0.06 |
| **Dead water level (m)** | 18.5 | 21.0 | 3.9 |
| **Normal water level (m)** | 30.1 | 29.8 | 12.5 |
| **Area (km^2^)** | 5.4 | 6.5 | 5.7 |
| **Total reservoir inflow (10^6^ m^3^)** | 82.3 | 278.1 | 260.6 |
| **Mean annual reservoir inflow (10^6^ m^3^/year)** | 41.2 | 30.9 | 29.0 |

DH: Donghu Reservoir, DT: Datun Reservoir, SWC: Shuangwangcheng Reservoir

The calculation periods for the inflow volume of Donghu Reservoir, Datun Reservoir, and Shuangwangcheng Reservoir are 2020–2022, 2013–2022, and 2013–2022, respectively

Table S3 Composition, relative importance index, and ecological guilds of fish assemblages detected from the three terminal reservoirs of the Eastern Route of the South-to-North Water Transfer Project

| Species | Code |  | | DH |  |  |  |  | DT |  |  |  |  | SWC |  |  |  | Ecological guilds |  |
| --- | --- | --- | --- | --- | --- | --- | --- | --- | --- | --- | --- | --- | --- | --- | --- | --- | --- | --- | --- |
|  |  | Total | | July | October | January | May | Total | July | October | January | May | Total | July | October | January | May |  |  |
| Salmoniformes |  |  | |  |  |  |  |  |  |  |  |  |  |  |  |  |  |  |  |
| Salangidae |  |  | |  |  |  |  |  |  |  |  |  |  |  |  |  |  |  |  |
| *Protosalanx hyalocranius* ( Abbott, 1901 ) ^*^ | *Phy* | 2.25 | | 23.51 | - | - | - | - | - | - | - | - | 103.71 | - | 1548.00 | - | - | Li, Ca, Mu, Dem ^b, c, d^ |  |
| *Neosalanx taihuensis* Chen, 1956 ^*^ | *Nta* | - | | - | - | - | - | - | - | - | - | - | 23.067 | - | - | 426.46 | - | Li, Pi, Mu, Dem ^b, c, d^ |  |
| Cypriniformes |  |  | |  |  |  |  |  |  |  |  |  |  |  |  |  |  |  |  |
| Cyprinidae |  |  | |  |  |  |  |  |  |  |  |  |  |  |  |  |  |  |  |
| *Cyprinus carpio* Linnaeus, 1758 | *Cca* | 309.24 | | 41.55 | - | 1927.00 | - | 32.97 | - | - | - | 1003.41 | 870.21 | - | - | 534.13 | 5604.26 | Eu, Om, De, Ad ^a, b, d^ |  |
| *Carassius auratus* (Linnaeus, 1758) | *Cau* | 277.34 | | 289.68 | 2005.67 | - | - | 1480.76 | 2816.19 | 832.69 | 1161.38 | 2837.18 | 4278.14 | 5259.97 | - | 11238.11 | 2181.9 | Eu, Om, De, Ad ^a, b, d^ |  |
| *Pseudorasbora parva* (Temminck & Schlegel, 1846) ^*^ | *Ppa* | 516.96 | | 1319.11 | 162.51 | - | 61.86 | 1215.66 | 373.61 | 1086.7 | - | 5050.88 | - | - | - | - | - | Li, Om, De, Ad ^a, b, d^ |  |
| *Abbottina rivularis* (Basilewsky, 1855) ^*^ | *Ari* | 5.82 | | 47.67 | - | - | - | 466.57 | 30.64 | 2126.08 | 55.02 | - | 719.74 | - | 5416.56 | 212.98 | - | Eu, Om, De, Ad ^a, b, d^ |  |
| *Sarcocheilichthys nigripinnis* (Günther, 1873) ^*^ | *Sni* | 671.76 | | 1645.73 | 167.38 | - | 469.19 | - | - | - | - | - | 11.85 | - | - | - | 164.48 | Eu, Om, De, Ad ^b, c, d^ |  |
| *Hemibarbus maculatus* Bleeker, 1871 | *Hma* | 45.50 | | - | 338.23 | - | 169.35 | 2701.23 | 1273.18 | 2078.95 | 5018.27 | 443.16 | 1479.96 | 527.11 | 550.04 | 1004.92 | 3364.13 | Rh, Om, Ml, Ad ^b, c, d^ |  |
| *Squaliobarbus curriculus* (Richardson, 1846) | *Scu* | - | | - | - | - | - | 85.35 | - | - | 718.53 | - | - | - | - | - | - | Eu, Om, Mu, De ^b, c^ |  |
| *Ctenopharyngodon idellus* (Valenciennes, 1844) | *Cid* | - | | - | - | - | - | 18.69 | - | - | - | 407.59 | - | - | - | - | - | Po, He, Ml, Dr ^a, b, d^ |  |
| *Toxabramis swinhonis* Günther, 1873 ^*^ | *Tsw* | - | | - | - | - | - | 1393.91 | 5741.98 | 1388.32 | - | 42.37 | - | - | - | - | - | Li, Pi, Mu, Dr ^a, b, d^ |  |
| *Parabramis pekinensis* (Basilewsky, 1855) | *Ppe* | - | | - | - | - | - | 737.32 | 2501.88 | - | 271.51 | 2890.57 | - | - | - | - | - | Rh, He, Ml, Dr ^a, b, d^ |  |
| *Megalobrama amblycephala* Yih, 1955 | *Mam* | - | | - | - | - | - | 620.53 | - | - | 5034.71 | - | 51.90 | - | - | 499.42 | - | Li, He, Ml, Ad ^a, b, d^ |  |
| *Hemiculter bleekeri* Warpachowski, 1888 ^*^ | *Hbl* | 11.43 | | 155.42 | - | - | - | 2.62 | 38.82 | - | - | - | - | - | - | - | - | Rh, Om, Mu, Dr ^b, c, d^ |  |
| *Hemiculter leucisculus* (Basilewsky, 1855) ^*^ | *Hle* | 2850.17 | | 2725.04 | 3432.29 | 408.61 | 11184.51 | 1743.83 | 2926.52 | 490.55 | 4952.68 | 244.06 | 362.23 | 70.33 | - | 852.76 | 854.35 | Li, Om, Mu Ad ^a, b, d^ |  |
| *Cultrichthys erythropterus* (Basilewsky, 1855) | *Cer* | 874.43 | | 1695.86 | 5029.02 | - | - | 134.52 | 325.8 | 172.73 | - | 349.38 | - | - | - | - | - | Li, Ca, Mu, Ad ^b, c, d^ |  |
| *Culter alburnus* Basilewsky, 1855 | *Cal* | 49.73 | | - | - | 339.39 | 57.18 | 116.8 | - | - | 1412.16 | - | 50.63 | - | - | 490.37 | - | Eu, Ca, Mu, Ad ^b, c, d^ |  |
| *Pseudolaubuca engraulis* (Nichols, 1925) ^*^ | *Pen* | - | | - | - | - | - | - | - | - | - | - | 24.50 | 78.61 | - | - | - | Eu, Pi, Mu, Dr ^b, c^ |  |
| *Pseudobrama simony* (Bleeker, 1864) ^*^ | *Psi* | 379.83 | | 2046.55 | 711.67 | - | - | 1462.66 | 640.57 | 6980.3 | 64.51 | 1303.69 | 15.22 | - | 526.96 | - | - | Rh, Pi, Mu, Dr ^a, c, d^ |  |
| *Acheilognathus macropterus* (Bleeker, 1871) ^*^ | *Ama* | 708.62 | | - | 5004.02 | - | 387.14 | 27.09 | - | 33.03 | 276.2 | - | 93.73 | - | 591.00 | - | 159.41 | Eu, Om, De, Sp ^a, b, d^ |  |
| *Acheilognathus chankaensis* (Dybowski, 1872) ^*^ | *Ach* | 364.97 | | 3577.41 | - | - | - | - | - | - | - | - | - | - | - | - | - | Eu, Om, De, Sp ^a, b, d^ |  |
| *Paracheilognathus imberbis* Günther, 1868 ^*^ | *Pim* | 205.98 | | 412.37 | 208.43 | - | 140.67 | 71.61 | 152.86 | 143.22 | - | 38.97 | 11.58 | 18.28 | - | - | - | Eu, Om, De, Sp ^b, c^ |  |
| *Rhodeus sinensis* Günther, 1868 ^*^ | *Rsi* | - | | - | - | - | - | 17.29 | - | - | - | 285.36 | - | - | - | - | - | Eu, Om, De, Sp ^b, c, d^ |  |
| *Rhodeus ocellatus* (Kner, 1866) ^*^ | *Roc* | - | | - | - | - | - | 2.21 | - | - | - | 37.17 | - | - | - | - | - | Eu, Om, De, Sp ^b, c, d^ |  |
| *Aristichthys nobilis* (Richardson, 1845) | *Hno* | 3829.08 | | 2329.35 | 644.6 | 13027.87 | - | 5.39 | - | - | - | 145.05 | 577.16 | - | - | 4222.97 | - | Po, Pi, Mu, Dr ^a, b, d^ |  |
| *Hypophthalmichthys molitrix* (Valenciennes, 1844) | *Hmo* | 1605.37 | | 3250.54 | 219.28 | 3840.85 | - | 127.97 | - | - | 978.99 | - | - | - | - | - | - | Po, Pi, Mu, Dr ^a, b, d^ |  |
| Cobitidae |  |  | |  |  |  |  |  |  |  |  |  |  |  |  |  |  |  |  |
| *Misgurnus anguillicaudatus* (Cantor, 1842) ^*^ | *Man* | 3.62 | | - | - | - | 230.06 | 2.83 | - | - | - | 58.48 | - | - | - | - | - | Li, Om, De, Ad ^b, c, d^ |  |
| Siluriformes |  |  | |  |  |  |  |  |  |  |  |  |  |  |  |  |  |  |  |
| Bagridae |  |  | |  |  |  |  |  |  |  |  |  |  |  |  |  |  |  |  |
| *Pelteobagrus fulvidraco* (Richardson, 1846) | *Pfu* | 388.90 | | 90.06 | 227.49 | 456.27 | 4623.75 | 125.93 | - | 361.57 | - | 1651.65 | 941.54 | - | 976.63 | 517.87 | 3375.09 | Eu, Ca, De, Ad ^a, b, d^ |  |
| *Pelteobagrus vachelli* (Richardson, 1846) | *Pva* | - | | - | - | - | - | 92.33 | 1570.69 | - | - | - | 430.36 | 4558.07 | - | - | - | Eu, Ca, De, Ad ^b, c^ |  |
| *Leiocassis argentivittatus* (Regan, 1905) ^*^ | *Lar* | 1.92 | | 15.30 | - | - | - | - | - | - | - | - | - | - | - | - | - | Eu, Ca, De, Ad ^b, c^ |  |
| Siluridae |  |  | |  |  |  |  |  |  |  |  |  |  |  |  |  |  |  |  |
| *Silurus asotus* Linnaeus, 1758 | *Sas* | 237.34 | | 271.11 | 581.35 | - | 1943.30 | 26.85 | 458.35 | - | - | - | 105.55 | - | 6404.19 | - | - | Rh, Ca, De, Ad ^b, c, d^ |  |
| Perciformes |  |  | |  |  |  |  |  |  |  |  |  |  |  |  |  |  |  |  |
| Eleotridae |  |  | |  |  |  |  |  |  |  |  |  |  |  |  |  |  |  |  |
| *Micropercops swinhonis* (Günther, 1873) ^*^ | *Msw* | - | | - | - | - | - | 2.16 | - | - | - | 35.75 | - | - | - | - | - | Eu, Om, De, Ad ^c, d^ |  |
| Gobiidae |  |  | |  |  |  |  |  |  |  |  |  |  |  |  |  |  |  |  |
| *Rhinogobius giurinus* (Rutter, 1897) ^*^ | *Rgi* | 28.15 | | - | 25.91 | - | 393.69 | 770.14 | 1118.80 | 291.25 | - | 2589.33 | 732.25 | 105.58 | 171.74 | - | 2717.13 | Eu, Ca, De, Ad ^b, c, d^ |  |
| *Tridentiger bifasciatus* Steindachner, 1881 ^*^ | *Tbi* | 324.94 | | 63.74 | 1242.14 | - | 339.30 | 1303.27 | 30.12 | 4014.61 | 56.04 | 585.95 | 624.51 | 82.77 | 1071.40 | - | 1121.53 | Eu, Ca, De, Ad ^b, c, e^ |  |
| Ophiocephalidae | ­­­­­ | |  |  |  |  |  |  |  |  |  |  |  |  |  |  |  |  |  |
| *Channa argus* (Cantor, 1842) | *Car* | - | | - | - | - | - | - | - | - | - | - | 139.48 | - | 2743.47 | - | 457.72 | Li, Ca, De, Pe ^a, b, d^ |  |
| Number of specie*s* |  | 23 | | 18 | 15 | 6 | 12 | 28 | 15 | 13 | 12 | 19 | 21 | 8 | 10 | 10 | 10 |  |  |

The relative importance index (IRI) (Pinkas et al., 1971): dominant species: IRI ≥ 500, important species: 100 ≤ IRI < 500, common species: 10 ≤ IRI < 100, rare species: IRI < 10

Eu: eurytopic, Rh: rheophilic, Li: limnophilic, Po: potamodromous (Feng et al., 2023); *: small- sized fish (Kopf et al., 2011); Ca: carnivorous, He: herbivorous, Om: omnivorous, Pi: planktivorous (Cheng and Zhou, 1997; Chen, 1998; Ni and Wu, 2006, Yan et al., 2023); Mu: middle and upper, Ml: middle and lower, De: demersal (Cheng and Zhou, 1997; Chen, 1998; Ni and Wu, 2006, Yan et al., 2023); Ad: adhesive eggs, Dem: demersal eggs, Pe: pelagic eggs, Dr: drifting eggs, Sp: spawning in mussels (Cheng and Zhou, 1997; Chen, 1998; Ni and Wu, 2006)

DH: Donghu Reservoir, DT: Datun Reservoir, SWC: Shuangwangcheng Reservoir. DH: Donghu Reservoir, DT: Datun Reservoir, SWC: Shuangwangcheng Reservoir

The lower case letter “a” indicates referring to Cheng and Zhou (1997), “b” indicates referring to Chen (1998), “c” indicates referring to Ni and Wu (2006), “d” indicates referring to Feng et al. (2023), and “e” indicates referring to Yan et al. (2023)

Chen, Q., Zhang, J., Chen, Y., Mo, K., Wang, J., Tang, L., Lin, Y., Chen, L., Gao, Y., Jiang, W., Zhang, Y., 2021. Inducing Flow Velocities to Manage Fish Reproduction in Regulated Rivers. Engineering 7, 178-186. 10.1016/j.eng.2020.06.013.

Cheng Q.T., Zhou C.W., 1997. The Fishes of Shandong Province. Shandong Science and Technology Press, Jinan.

Feng, K., Deng, W., Li, H., Guo, Q., Tao, K., Yuan, J., Liu, J., Li, Z., Lek, S., Hugueny, B., Wang, Q., 2023. Direct and indirect effects of a fishing ban on lacustrine fish community do not result in a full recovery. Journal of Applied Ecology 60, 2210-2222. https://doi.org/10.1111/1365-2664.14491.

Kopf, R.K., Shaw, C., Humphries, P., 2017. Trait-based prediction of extinction risk of small-bodied freshwater fishes. Conservation Biology 31, 581-591. https://doi.org/10.1111/cobi.12882.

Ni, Y., Wu, H.L., 2006. Fishes of Jiangsu Province. China Agriculture Press, Beijing.

Pinkas, L., Oliphant, M.S., Iverson, I.L.K., 1971. Food habits of albacore, bluefin tuna, and bonito in Californian waters. Fish Bulletin 152, 11–105.

Yan, H., Chen, S., Liu, X., Cheng, Z., Schmidt, B.V., He, W., Cheng, F., Xie, S., 2023. Investigations of Fish Assemblages Using Two Methods in Three Terminal Reservoirs of the East Route of South-to-North Water Transfer Project, China. Animals 13, 1614. https://doi.org/10.3390/ani13101614.

Table S4 Alpha functional traits of fish species

| **Traits** | **Type** | **Units / description** | **Ecological meaning** |
| --- | --- | --- | --- |
| **Maximum TL** | Continuous | Maximum total length (mm) | Reflects position in the food web, metabolic rates, dispersal ability, mobility and home range |
| **Feeding** | Categorical | Planktivorous, Omnivorous, Carnivorous, Herbivorous | Relates to position in the food web, influences on the abundance of other species, and adaptations to habitat |
| **Trophic Level** | Continuous | Trophic position in the food chain | Describes the position in the food web, food acquisition ability, and nutrient budget |
| **Migration type** | Categorical | Sedentary, Migratory, Semi-migratory | Reflects the physiological ability to deal with pressure and conditions of hydrology |
| **Position of mouth** | Categorical | Terminal, Upper, Lower | Reflects feeding strategy and trophic group |
| **Eye size** | Continuous | Head height (mm) / Eye diameter (mm) | Reflects the sensitivity to stimulation, food acquisition, and defense against predation |
| **Spawning type** | Categorical | Adhesive eggs, Demersal eggs, Pelagic eggs, Drifting eggs, Spawning in mussels | Reflects the development mode and success rate of fish fertilized eggs in the water body |
| **Maturity age** | Continuous | Age at maturation (years) | Describes the physiological ability to deal with reproduction. Relates with stability of populations over time |
| **Maturity TL** | Continuous | Total length at maturation (mm) | Relates to reproductive mode, defense against predation, and stability of populations over time |
| **Longevity** | Continuous | Maximum life span (years) | Describes the longevity of individuals. Relates to stability of populations overtime |
| **Body shape** | Categorical | Compressed, Oval, Anguilliform, Dorso-ventrally flattened, Cylindrical | Reflects the physiological ability to deal with mobility, food acquisition, and defense against predation |
| **Inhabiting water** **layers** | Categorical | Middle and upper, Middle and lower, Demersal | Reflects the niche of fish in the water, it effects on the acquisition of potential prey and the transfer of nutrients between vertical aquifers |

The functional traits of each species were identified based on published literature (Cheng and Zhou, 1997; Chen, 1998; Ni and Wu, 2006; Feng et al., 2023) and FishBase (www.fishbase.org/)

Chen, Q., Zhang, J., Chen, Y., Mo, K., Wang, J., Tang, L., Lin, Y., Chen, L., Gao, Y., Jiang, W., Zhang, Y., 2021. Inducing Flow Velocities to Manage Fish Reproduction in Regulated Rivers. Engineering 7, 178-186. 10.1016/j.eng.2020.06.013.

Cheng Q.T., Zhou C.W., 1997. The Fishes of Shandong Province. Shandong Science and Technology Press, Jinan.

Feng, K., Deng, W., Li, H., Guo, Q., Tao, K., Yuan, J., Liu, J., Li, Z., Lek, S., Hugueny, B., Wang, Q., 2023. Direct and indirect effects of a fishing ban on lacustrine fish community do not result in a full recovery. Journal of Applied Ecology 60, 2210-2222. https://doi.org/10.1111/1365-2664.14491.

Ni, Y., Wu, H.L., 2006. Fishes of Jiangsu Province. China Agriculture Press, Beijing.

Table S5 Bray-Curtis dissimilarity matrix among fish assemblages in the three terminal reservoirs of the Eastern Route of the South-to-North Water Transfer Project

|  | **DH7** | **DH10** | **DH1** | **DH5** | **DT7** | **DT10** | **DT1** | **DT5** | **SWC7** | **SWC10** | **SWC1** | **SWC5** |
| --- | --- | --- | --- | --- | --- | --- | --- | --- | --- | --- | --- | --- |
| **DH7** | 0 |  |  |  |  |  |  |  |  |  |  |  |
| **DH10** | 0.486 | 0 |  |  |  |  |  |  |  |  |  |  |
| **DH1** | 0.798 | 0.794 | 0 |  |  |  |  |  |  |  |  |  |
| **DH5** | 0.696 | 0.491 | 0.841 | 0 |  |  |  |  |  |  |  |  |
| **DT7** | 0.614 | 0.520 | 0.959 | 0.616 | 0 |  |  |  |  |  |  |  |
| **DT10** | 0.629 | 0.557 | 0.931 | 0.693 | 0.496 | 0 |  |  |  |  |  |  |
| **DT1** | 0.746 | 0.604 | 0.852 | 0.584 | 0.580 | 0.749 | 0 |  |  |  |  |  |
| **DT5** | 0.624 | 0.603 | 0.836 | 0.692 | 0.524 | 0.504 | 0.792 | 0 |  |  |  |  |
| **SWC7** | 0.867 | 0.768 | 0.936 | 0.722 | 0.659 | 0.743 | 0.776 | 0.736 | 0 |  |  |  |
| **SWC10** | 0.841 | 0.730 | 0.920 | 0.635 | 0.809 | 0.648 | 0.788 | 0.806 | 0.773 | 0 |  |  |
| **SWC1** | 0.824 | 0.710 | 0.632 | 0.786 | 0.770 | 0.779 | 0.619 | 0.754 | 0.702 | 0.798 | 0 |  |
| **SWC5** | 0.823 | 0.655 | 0.741 | 0.503 | 0.687 | 0.633 | 0.695 | 0.580 | 0.582 | 0.605 | 0.580 | 0 |

Values represent pairwise dissimilarities (0 = identical, 1 = completely dissimilar). The symbol includes the sampling site and month; for example, DH1 indicates sampling at Donghu Reservoir in January

Table S6 Top six species contributing to the dissimilarity of the fish assemblage in the three terminal reservoirs of the Eastern Route of the South-to-North Water Transfer Project

| **Species** | **DT-SWC** | | **DH-SWC** | | **DH - DT** | |
| --- | --- | --- | --- | --- | --- | --- |
|  | **Average dissimilarity** | **Contribution (%)** | **Average dissimilarity** | **Contribution (%)** | **Average dissimilarity** | **Contribution (%)** |
| ***Pseudorasbora parva*** | 7.23 | 10.07 | 3.30 | 4.36 | 5.14 | 7.44 |
| ***Hemiculter leucisculus*** | 7.07 | 9.85 | 9.30 | 12.31 | 4.82 | 6.97 |
| ***Toxabramis swinhonis*** | 6.34 | 8.83 | - | - | 4.82 | 6.98 |
| ***Pseudobrama simony*** | 5.33 | 7.42 | 2.83 | 3.75 | 3.86 | 5.58 |
| ***Tridentiger bifasciatus*** | 5.22 | 7.27 | 2.71 | 3.59 | 4.19 | 6.06 |
| ***Rhinogobius giurinus*** | 5.01 | 6.98 | 3.34 | 4.41 | 4.20 | 6.08 |
| **Total** |  | 50.42 |  | 28.42 |  | 39.11 |

DH: Donghu Reservoir, DT: Datun Reservoir, SWC: Shuangwangcheng Reservoir

Table S7 The distance matrix accounting for the total dissimilarity of beta taxonomic diversity detected from the three terminal reservoirs of the Eastern Route of the South-to-North Water Transfer Project

|  | **DH7** | **DH10** | **DH1** | **DH5** | **DT7** | **DT10** | **DT1** | **DT5** | **SWC7** | **SWC10** | **SWC1** | **SWC5** |
| --- | --- | --- | --- | --- | --- | --- | --- | --- | --- | --- | --- | --- |
| **DH7** | 0 |  |  |  |  |  |  |  |  |  |  |  |
| **DH10** | 0.273 | 0 |  |  |  |  |  |  |  |  |  |  |
| **DH1** | 0.583 | 0.619 | 0 |  |  |  |  |  |  |  |  |  |
| **DH5** | 0.533 | 0.259 | 0.667 | 0 |  |  |  |  |  |  |  |  |
| **DT7** | 0.394 | 0.333 | 0.905 | 0.481 | 0 |  |  |  |  |  |  |  |
| **DT10** | 0.419 | 0.214 | 0.789 | 0.360 | 0.214 | 0 |  |  |  |  |  |  |
| **DT1** | 0.600 | 0.481 | 0.667 | 0.583 | 0.481 | 0.440 | 0 |  |  |  |  |  |
| **DT5** | 0.459 | 0.353 | 0.680 | 0.484 | 0.353 | 0.313 | 0.613 | 0 |  |  |  |  |
| **SWC7** | 0.692 | 0.478 | 0.857 | 0.500 | 0.391 | 0.429 | 0.600 | 0.556 | 0 |  |  |  |
| **SWC10** | 0.571 | 0.440 | 0.875 | 0.455 | 0.520 | 0.391 | 0.545 | 0.655 | 0.667 | 0 |  |  |
| **SWC1** | 0.571 | 0.600 | 0.375 | 0.636 | 0.680 | 0.565 | 0.455 | 0.586 | 0.667 | 0.700 | 0 |  |
| **SWC5** | 0.571 | 0.360 | 0.625 | 0.364 | 0.600 | 0.391 | 0.545 | 0.517 | 0.444 | 0.400 | 0.500 | 0 |

Values represent pairwise dissimilarities (0 = identical, 1 = completely dissimilar). The symbol includes the sampling site and month; for example, DH1 indicates sampling at Donghu Reservoir in January

Table S8 The distance matrix accounting for the turnover component of beta taxonomic diversity detected from the three terminal reservoirs of the Eastern Route of the South-to-North Water Transfer Project

|  | **DH7** | **DH10** | **DH1** | **DH5** | **DT7** | **DT10** | **DT1** | **DT5** | **SWC7** | **SWC10** | **SWC1** | **SWC5** |
| --- | --- | --- | --- | --- | --- | --- | --- | --- | --- | --- | --- | --- |
| **DH7** | 0 |  |  |  |  |  |  |  |  |  |  |  |
| **DH10** | 0.200 | 0 |  |  |  |  |  |  |  |  |  |  |
| **DH1** | 0.167 | 0.333 | 0 |  |  |  |  |  |  |  |  |  |
| **DH5** | 0.417 | 0.167 | 0.500 | 0 |  |  |  |  |  |  |  |  |
| **DT7** | 0.333 | 0.333 | 0.833 | 0.417 | 0 |  |  |  |  |  |  |  |
| **DT10** | 0.308 | 0.154 | 0.667 | 0.333 | 0.154 | 0 |  |  |  |  |  |  |
| **DT1** | 0.500 | 0.417 | 0.500 | 0.583 | 0.417 | 0.417 | 0 |  |  |  |  |  |
| **DT5** | 0.444 | 0.267 | 0.333 | 0.333 | 0.267 | 0.154 | 0.500 | 0 |  |  |  |  |
| **SWC7** | 0.500 | 0.250 | 0.833 | 0.375 | 0.125 | 0.250 | 0.500 | 0.250 | 0 |  |  |  |
| **SWC10** | 0.400 | 0.300 | 0.833 | 0.400 | 0.400 | 0.300 | 0.500 | 0.500 | 0.625 | 0 |  |  |
| **SWC1** | 0.400 | 0.500 | 0.167 | 0.600 | 0.600 | 0.500 | 0.400 | 0.400 | 0.625 | 0.700 | 0 |  |
| **SWC5** | 0.400 | 0.200 | 0.500 | 0.300 | 0.500 | 0.300 | 0.500 | 0.300 | 0.375 | 0.400 | 0.500 | 0 |

Values represent pairwise dissimilarities (0 = identical, 1 = completely dissimilar). The symbol includes the sampling site and month; for example, DH1 indicates sampling at Donghu Reservoir in January

Table S9 The distance matrix accounting for the nestedness component of beta taxonomic diversity detected from the three terminal reservoirs of the Eastern Route of the South-to-North Water Transfer Project

|  | **DH7** | **DH10** | **DH1** | **DH5** | **DT7** | **DT10** | **DT1** | **DT5** | **SWC7** | **SWC10** | **SWC1** | **SWC5** |
| --- | --- | --- | --- | --- | --- | --- | --- | --- | --- | --- | --- | --- |
| **DH7** | 0 |  |  |  |  |  |  |  |  |  |  |  |
| **DH10** | 0.396 | 0 |  |  |  |  |  |  |  |  |  |  |
| **DH1** | 0.935 | 0.882 | 0 |  |  |  |  |  |  |  |  |  |
| **DH5** | 0.776 | 0.589 | 0.957 | 0 |  |  |  |  |  |  |  |  |
| **DT7** | 0.570 | 0.335 | 0.922 | 0.598 | 0 |  |  |  |  |  |  |  |
| **DT10** | 0.801 | 0.597 | 0.981 | 0.491 | 0.500 | 0 |  |  |  |  |  |  |
| **DT1** | 0.563 | 0.345 | 0.883 | 0.769 | 0.366 | 0.636 | 0 |  |  |  |  |  |
| **DT5** | 0.480 | 0.394 | 0.923 | 0.790 | 0.533 | 0.755 | 0.440 | 0 |  |  |  |  |
| **SWC7** | 0.948 | 0.897 | 0.970 | 0.799 | 0.833 | 0.741 | 0.871 | 0.938 | 0 |  |  |  |
| **SWC10** | 0.595 | 0.653 | 0.980 | 0.722 | 0.603 | 0.644 | 0.751 | 0.748 | 0.928 | 0 |  |  |
| **SWC1** | 0.650 | 0.596 | 0.752 | 0.807 | 0.631 | 0.775 | 0.602 | 0.685 | 0.896 | 0.741 | 0 |  |
| **SWC5** | 0.895 | 0.811 | 1.000 | 0.578 | 0.784 | 0.687 | 0.835 | 0.877 | 0.774 | 0.790 | 0.887 | 0 |

Values represent pairwise dissimilarities (0 = identical, 1 = completely dissimilar). The symbol includes the sampling site and month; for example, DH1 indicates sampling at Donghu Reservoir in January

Table S10 The distance matrix accounting for the total dissimilarity of beta functional diversity detected from the three terminal reservoirs of the Eastern Route of the South-to-North Water Transfer Project

|  | **DH7** | **DH10** | **DH1** | **DH5** | **DT7** | **DT10** | **DT1** | **DT5** | **SWC7** | **SWC10** | **SWC1** | **SWC5** |
| --- | --- | --- | --- | --- | --- | --- | --- | --- | --- | --- | --- | --- |
| **DH7** | 0 |  |  |  |  |  |  |  |  |  |  |  |
| **DH10** | 0.396 | 0 |  |  |  |  |  |  |  |  |  |  |
| **DH1** | 0.935 | 0.882 | 0 |  |  |  |  |  |  |  |  |  |
| **DH5** | 0.776 | 0.589 | 0.957 | 0 |  |  |  |  |  |  |  |  |
| **DT7** | 0.570 | 0.335 | 0.922 | 0.598 | 0 |  |  |  |  |  |  |  |
| **DT10** | 0.801 | 0.597 | 0.981 | 0.491 | 0.500 | 0 |  |  |  |  |  |  |
| **DT1** | 0.563 | 0.345 | 0.883 | 0.769 | 0.366 | 0.636 | 0 |  |  |  |  |  |
| **DT5** | 0.480 | 0.394 | 0.923 | 0.790 | 0.533 | 0.755 | 0.440 | 0 |  |  |  |  |
| **SWC7** | 0.948 | 0.897 | 0.970 | 0.799 | 0.833 | 0.741 | 0.871 | 0.938 | 0 |  |  |  |
| **SWC10** | 0.595 | 0.653 | 0.980 | 0.722 | 0.603 | 0.644 | 0.751 | 0.748 | 0.928 | 0 |  |  |
| **SWC1** | 0.650 | 0.596 | 0.752 | 0.807 | 0.631 | 0.775 | 0.602 | 0.685 | 0.896 | 0.741 | 0 |  |
| **SWC5** | 0.895 | 0.811 | 1.000 | 0.578 | 0.784 | 0.687 | 0.835 | 0.877 | 0.774 | 0.790 | 0.887 | 0 |

Values represent pairwise dissimilarities (0 = identical, 1 = completely dissimilar). The symbol includes the sampling site and month; for example, DH1 indicates sampling at Donghu Reservoir in January

Table S11 The distance matrix accounting for the turnover component of beta functional diversity detected from the three terminal reservoirs of the Eastern Route of the South-to-North Water Transfer Project

|  | **DH7** | **DH10** | **DH1** | **DH5** | **DT7** | **DT10** | **DT1** | **DT5** | **SWC7** | **SWC10** | **SWC1** | **SWC5** |
| --- | --- | --- | --- | --- | --- | --- | --- | --- | --- | --- | --- | --- |
| **DH7** | 0 |  |  |  |  |  |  |  |  |  |  |  |
| **DH10** | 0.087 | 0 |  |  |  |  |  |  |  |  |  |  |
| **DH1** | 0.022 | 0.093 | 0 |  |  |  |  |  |  |  |  |  |
| **DH5** | 0.135 | 0.110 | 0.887 | 0 |  |  |  |  |  |  |  |  |
| **DT7** | 0.050 | 0.106 | 0.630 | 0.403 | 0 |  |  |  |  |  |  |  |
| **DT10** | 0.117 | 0.012 | 0.956 | 0.447 | 0.172 | 0 |  |  |  |  |  |  |
| **DT1** | 0.151 | 0.206 | 0.352 | 0.615 | 0.308 | 0.320 | 0 |  |  |  |  |  |
| **DT5** | 0.427 | 0.188 | 0.030 | 0.308 | 0.105 | 0.074 | 0.050 | 0 |  |  |  |  |
| **SWC7** | 0.006 | 0.000 | 0.966 | 0.342 | 0.000 | 0.257 | 0.099 | 0.000 | 0 |  |  |  |
| **SWC10** | 0.298 | 0.615 | 0.871 | 0.481 | 0.527 | 0.254 | 0.730 | 0.616 | 0.420 | 0 |  |  |
| **SWC1** | 0.093 | 0.379 | 0.018 | 0.748 | 0.589 | 0.676 | 0.512 | 0.296 | 0.484 | 0.651 | 0 |  |
| **SWC5** | 0.247 | 0.280 | 0.999 | 0.371 | 0.470 | 0.579 | 0.536 | 0.260 | 0.569 | 0.326 | 0.764 | 0 |

Values represent pairwise dissimilarities (0 = identical, 1 = completely dissimilar). The symbol includes the sampling site and month; for example, DH1 indicates sampling at Donghu Reservoir in January

Table S12 The distance matrix accounting for the nestedness component of beta functional diversity detected from the three terminal reservoirs of the Eastern Route of the South-to-North Water Transfer Project

|  | **DH7** | **DH10** | **DH1** | **DH5** | **DT7** | **DT10** | **DT1** | **DT5** | **SWC7** | **SWC10** | **SWC1** | **SWC5** |
| --- | --- | --- | --- | --- | --- | --- | --- | --- | --- | --- | --- | --- |
| **DH7** | 0 |  |  |  |  |  |  |  |  |  |  |  |
| **DH10** | 0.309 | 0 |  |  |  |  |  |  |  |  |  |  |
| **DH1** | 0.913 | 0.788 | 0 |  |  |  |  |  |  |  |  |  |
| **DH5** | 0.641 | 0.479 | 0.071 | 0 |  |  |  |  |  |  |  |  |
| **DT7** | 0.520 | 0.229 | 0.292 | 0.195 | 0 |  |  |  |  |  |  |  |
| **DT10** | 0.684 | 0.584 | 0.025 | 0.043 | 0.327 | 0 |  |  |  |  |  |  |
| **DT1** | 0.412 | 0.139 | 0.531 | 0.154 | 0.058 | 0.316 | 0 |  |  |  |  |  |
| **DT5** | 0.053 | 0.206 | 0.892 | 0.482 | 0.429 | 0.680 | 0.390 | 0 |  |  |  |  |
| **SWC7** | 0.942 | 0.897 | 0.004 | 0.457 | 0.833 | 0.484 | 0.772 | 0.937 | 0 |  |  |  |
| **SWC10** | 0.297 | 0.038 | 0.109 | 0.240 | 0.076 | 0.390 | 0.021 | 0.132 | 0.508 | 0 |  |  |
| **SWC1** | 0.557 | 0.216 | 0.734 | 0.059 | 0.042 | 0.099 | 0.090 | 0.390 | 0.412 | 0.090 | 0 |  |
| **SWC5** | 0.648 | 0.530 | 0.000 | 0.207 | 0.314 | 0.108 | 0.299 | 0.618 | 0.204 | 0.463 | 0.123 | 0 |

Values represent pairwise dissimilarities (0 = identical, 1 = completely dissimilar). The symbol includes the sampling site and month; for example, DH1 indicates sampling at Donghu Reservoir in January

Table S13 The distance matrix accounting for the total dissimilarity of beta phylogenetic diversity detected from the three terminal reservoirs of the Eastern Route of the South-to-North Water Transfer Project

|  | **DH7** | **DH10** | **DH1** | **DH5** | **DT7** | **DT10** | **DT1** | **DT5** | **SWC7** | **SWC10** | **SWC1** | **SWC5** |
| --- | --- | --- | --- | --- | --- | --- | --- | --- | --- | --- | --- | --- |
| **DH7** | 0 |  |  |  |  |  |  |  |  |  |  |  |
| **DH10** | 0.157 | 0 |  |  |  |  |  |  |  |  |  |  |
| **DH1** | 0.522 | 0.510 | 0 |  |  |  |  |  |  |  |  |  |
| **DH5** | 0.201 | 0.052 | 0.539 | 0 |  |  |  |  |  |  |  |  |
| **DT7** | 0.131 | 0.074 | 0.528 | 0.114 | 0 |  |  |  |  |  |  |  |
| **DT10** | 0.148 | 0.096 | 0.475 | 0.137 | 0.078 | 0 |  |  |  |  |  |  |
| **DT1** | 0.266 | 0.220 | 0.612 | 0.266 | 0.194 | 0.138 | 0 |  |  |  |  |  |
| **DT5** | 0.225 | 0.167 | 0.523 | 0.138 | 0.153 | 0.139 | 0.259 | 0 |  |  |  |  |
| **SWC7** | 0.217 | 0.129 | 0.479 | 0.151 | 0.095 | 0.097 | 0.185 | 0.160 | 0 |  |  |  |
| **SWC10** | 0.149 | 0.151 | 0.582 | 0.184 | 0.192 | 0.218 | 0.293 | 0.279 | 0.209 | 0 |  |  |
| **SWC1** | 0.341 | 0.504 | 0.273 | 0.522 | 0.464 | 0.419 | 0.520 | 0.484 | 0.454 | 0.409 | 0 |  |
| **SWC5** | 0.241 | 0.136 | 0.472 | 0.157 | 0.165 | 0.125 | 0.199 | 0.186 | 0.100 | 0.147 | 0.446 | 0 |

Values represent pairwise dissimilarities (0 = identical, 1 = completely dissimilar). The symbol includes the sampling site and month; for example, DH1 indicates sampling at Donghu Reservoir in January

Table S14 The distance matrix accounting for the turnover component of beta phylogenetic diversity detected from the three terminal reservoirs of the Eastern Route of the South-to-North Water Transfer Project

|  | **DH7** | **DH10** | **DH1** | **DH5** | **DT7** | **DT10** | **DT1** | **DT5** | **SWC7** | **SWC10** | **SWC1** | **SWC5** |
| --- | --- | --- | --- | --- | --- | --- | --- | --- | --- | --- | --- | --- |
| **DH7** | 0 |  |  |  |  |  |  |  |  |  |  |  |
| **DH10** | 0.087 | 0 |  |  |  |  |  |  |  |  |  |  |
| **DH1** | 0.006 | 0.091 | 0 |  |  |  |  |  |  |  |  |  |
| **DH5** | 0.157 | 0.029 | 0.113 | 0 |  |  |  |  |  |  |  |  |
| **DT7** | 0.075 | 0.058 | 0.102 | 0.107 | 0 |  |  |  |  |  |  |  |
| **DT10** | 0.045 | 0.066 | 0.070 | 0.086 | 0.031 | 0 |  |  |  |  |  |  |
| **DT1** | 0.084 | 0.109 | 0.396 | 0.139 | 0.062 | 0.050 | 0 |  |  |  |  |  |
| **DT5** | 0.219 | 0.105 | 0.019 | 0.097 | 0.106 | 0.044 | 0.084 | 0 |  |  |  |  |
| **SWC7** | 0.090 | 0.070 | 0.118 | 0.070 | 0.016 | 0.068 | 0.132 | 0.032 | 0 |  |  |  |
| **SWC10** | 0.122 | 0.110 | 0.169 | 0.166 | 0.167 | 0.152 | 0.149 | 0.262 | 0.112 | 0 |  |  |
| **SWC1** | 0.061 | 0.359 | 0.014 | 0.363 | 0.293 | 0.277 | 0.464 | 0.273 | 0.345 | 0.190 | 0 |  |
| **SWC5** | 0.159 | 0.117 | 0.049 | 0.117 | 0.133 | 0.115 | 0.107 | 0.106 | 0.059 | 0.085 | 0.302 | 0 |

Values represent pairwise dissimilarities (0 = identical, 1 = completely dissimilar). The symbol includes the sampling site and month; for example, DH1 indicates sampling at Donghu Reservoir in January

Table S15 The distance matrix accounting for the nestedness component of beta phylogenetic diversity detected from the three terminal reservoirs of the Eastern Route of the South-to-North Water Transfer Project

|  | **DH7** | **DH10** | **DH1** | **DH5** | **DT7** | **DT10** | **DT1** | **DT5** | **SWC7** | **SWC10** | **SWC1** | **SWC5** |
| --- | --- | --- | --- | --- | --- | --- | --- | --- | --- | --- | --- | --- |
| **DH7** | 0 |  |  |  |  |  |  |  |  |  |  |  |
| **DH10** | 0.070 | 0 |  |  |  |  |  |  |  |  |  |  |
| **DH1** | 0.517 | 0.419 | 0 |  |  |  |  |  |  |  |  |  |
| **DH5** | 0.045 | 0.023 | 0.426 | 0 |  |  |  |  |  |  |  |  |
| **DT7** | 0.055 | 0.016 | 0.426 | 0.006 | 0 |  |  |  |  |  |  |  |
| **DT10** | 0.103 | 0.030 | 0.406 | 0.051 | 0.047 | 0 |  |  |  |  |  |  |
| **DT1** | 0.182 | 0.110 | 0.216 | 0.127 | 0.132 | 0.088 | 0 |  |  |  |  |  |
| **DT5** | 0.006 | 0.061 | 0.504 | 0.040 | 0.046 | 0.096 | 0.175 | 0 |  |  |  |  |
| **SWC7** | 0.127 | 0.059 | 0.362 | 0.081 | 0.079 | 0.030 | 0.053 | 0.127 | 0 |  |  |  |
| **SWC10** | 0.027 | 0.041 | 0.413 | 0.019 | 0.025 | 0.066 | 0.144 | 0.017 | 0.097 | 0 |  |  |
| **SWC1** | 0.280 | 0.145 | 0.259 | 0.159 | 0.171 | 0.142 | 0.057 | 0.211 | 0.108 | 0.219 | 0 |  |
| **SWC5** | 0.082 | 0.018 | 0.423 | 0.039 | 0.033 | 0.010 | 0.092 | 0.080 | 0.040 | 0.061 | 0.144 | 0 |

Values represent pairwise dissimilarities (0 = identical, 1 = completely dissimilar). The symbol includes the sampling site and month; for example, DH1 indicates sampling at Donghu Reservoir in January


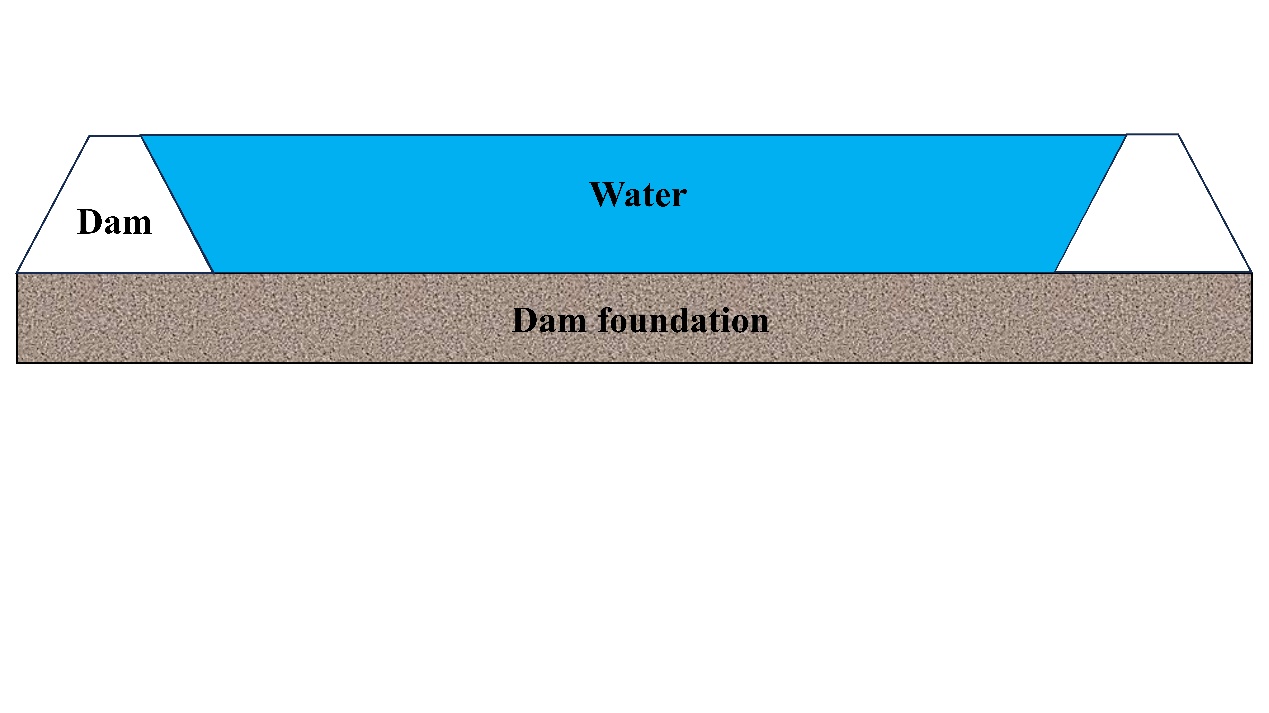
Figure S1 Morphological schematic of the three terminal reservoirs of the Eastern Route of the South-to-North Water Transfer Project

There is an island with an area of approximately 0.04 km² located only in Donghu Reservoir


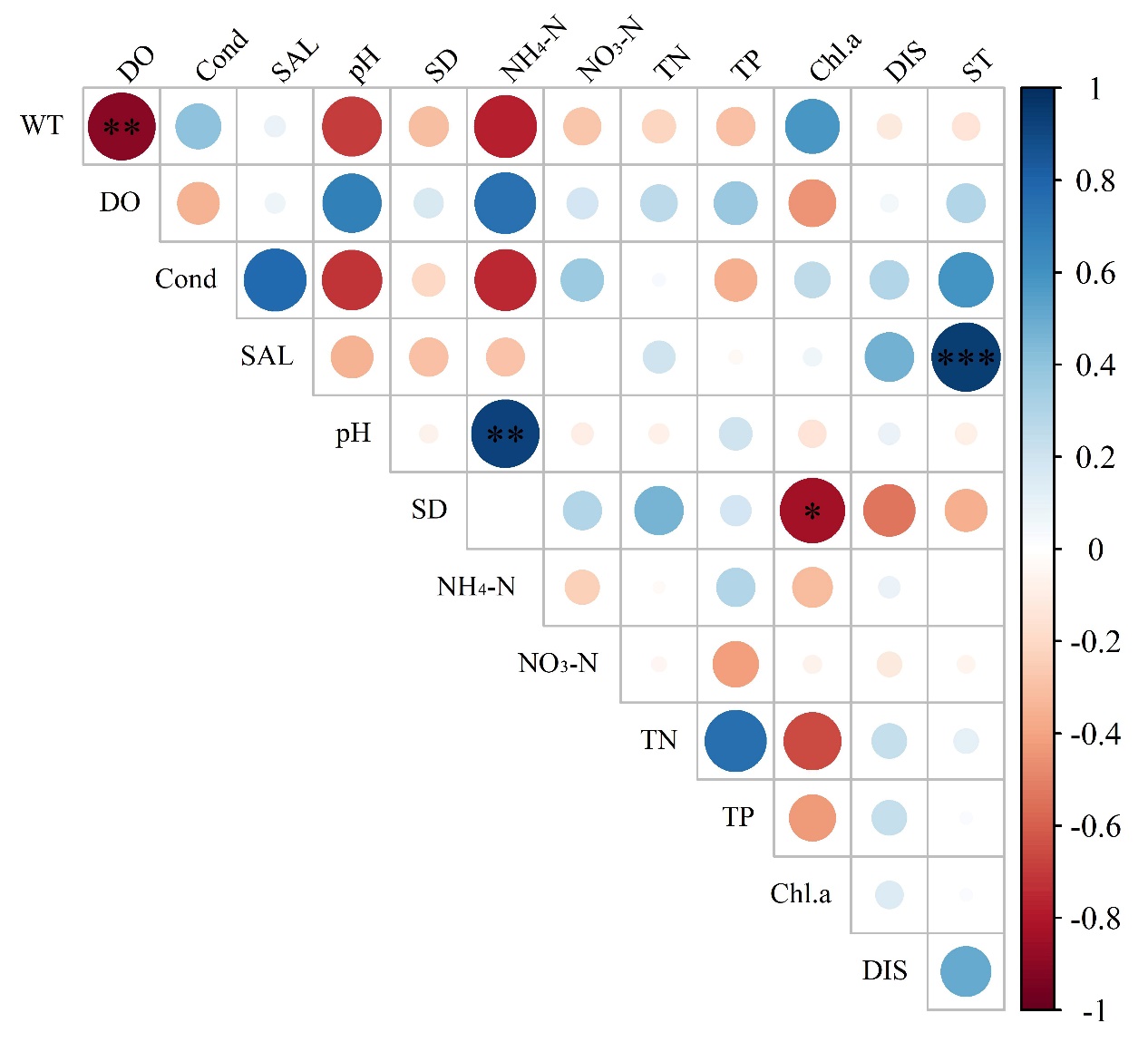


Figure S2 The Spearman’s correlations between the environmental factors in the study


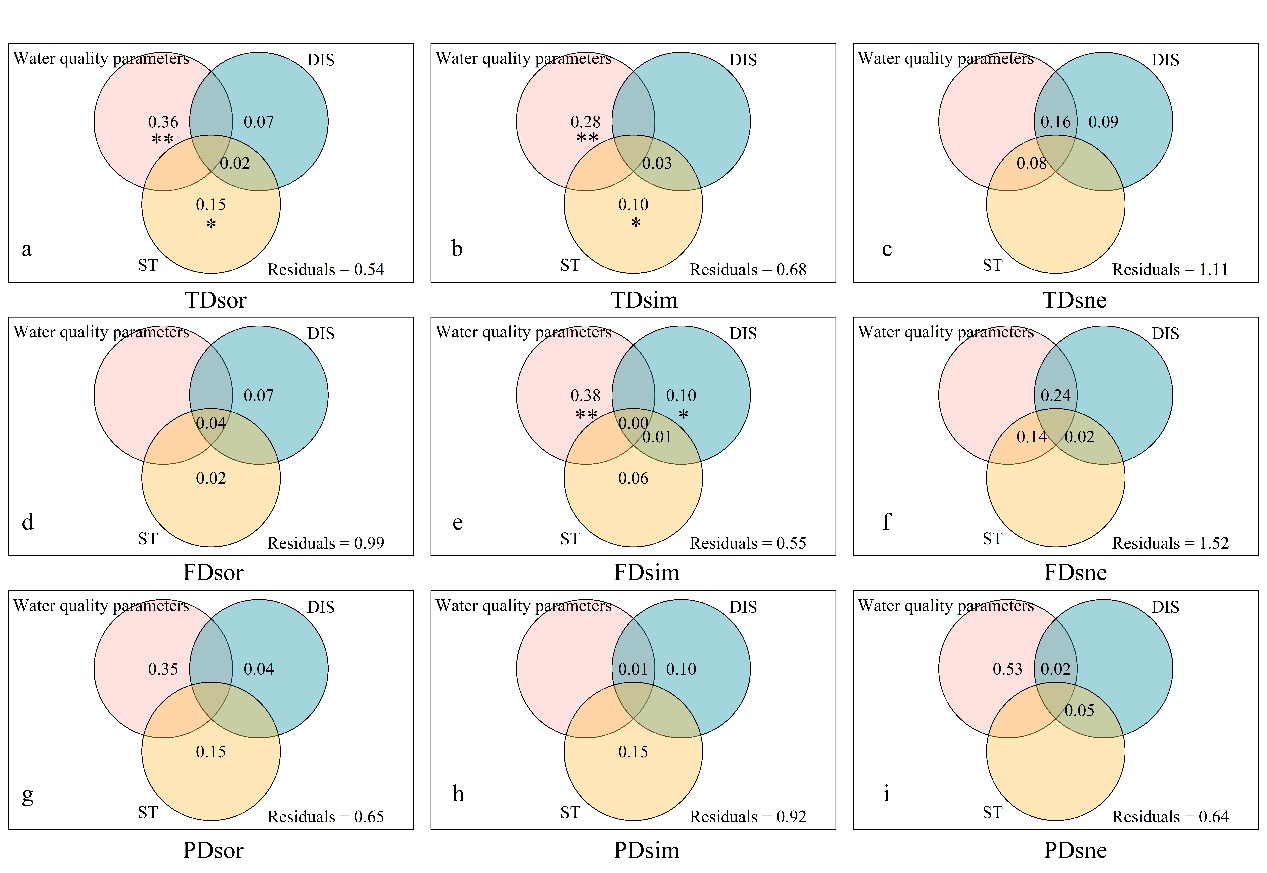


Figure S3 Variation partitioning analysis for fish taxonomic beta diversity, (a) TDsor; functional beta diversity, (d) Fdsor; and phylogenetic beta diversity, (g) PDsor; and their turnover aspects, (b) TDsim, (e) FDsim, (h) PDsim; nestedness aspects, (c)TDsne, (f) FDsne and (i) PDsne explained by environmental factors in the three terminal reservoirs of the Eastern Route of the South-to-North Water Transfer Project

Adjusted R^2^ value < 0 is not shown. * P < 0.05, ** P < 0.01
